# Pan-cancer analysis of super enhancer-induced PRR7-AS1 as a potential prognostic and immunological biomarker

**DOI:** 10.3389/fgene.2023.1160599

**Published:** 2023-04-06

**Authors:** Rui Wang, Na Liu, Guiqing Li, Jing Liu, Xiaolin Ma, Xinling Liu, Jiaqiu Li

**Affiliations:** ^1^ Department of Oncology, Affiliated Hospital of Weifang Medical University, School of Clinical Medicine, Weifang Medical University, Weifang, China; ^2^ Clinical Research Center, Affiliated Hospital of Weifang Medical University, Weifang, China

**Keywords:** PRR7-AS1, bioinformatics, pan-cancer, biomarker, tumor immunity, super enhancer

## Abstract

**Introduction:** Systematic pan-cancer analysis of the roles and regulatory mechanisms for PRR7-AS1 is currently not available.

**Methods:** In the present study, a comprehensive bioinformatic approach was used to mine the underlying oncogenic effects of PRR7-AS1, including expression status, prognostic value and immune characteristics.

**Results:** We discovered that PRR7-AS1 expression was remarkably upregulated in most cancer types and exhibited a negative correlation with the prognosis. Furthermore, PRR7-AS1 expression was inversely connected with the majority of tumor-infiltrating immune cells, immune scores and immune checkpoint gene expression in pancancer. There was also a significant correlation between PRR7-AS1 expression status and tumor mutational burden, microsatellite instability, and neoantigens in certain tumors. PRR7-AS1 had the best predictive power for immune checkpoint blockade efficacy compared to other well-recognized biomarkers. PRR7-AS1 overexpression could affect cytotoxic T cells-mediated antitumor responses. Functional enrichment analysis revealed that PRR7-AS1 might be involved in the metabolic pathways. Super enhancer activity might have participated in the regulation of PRR7-AS1 expression. And we constructed the competitive endogenous RNA networks for PRR7-AS1.

**Discussion:** In general, PRR7-AS1 had the potential to be a diagnostic, prognostic and immune biomarker for pan cancer. PRR7-AS1 was correlated with an immunosuppressive microenvironment and was a new potential target for immunotherapy. Epigenetic factors were the driving forces for PRR7-AS1 overexpression in tumors.

## Introduction

Cancer remains a serious increasing challenge for public health (Siegel et al., 2020). According to the World Health Organization (WHO) estimates, in 2020, cancer constituted the second deadliest factor globally (Sung et al., 2021). While surgical resection combined with chemoradiotherapy remains the most common first-line treatment for cancer, their utilization in clinical therapy is restricted attributed to the increasing drug resistance and severe side effects (Vasan et al., 2019; Herrmann, 2020). Over recent years, targeting the tumor microenvironment (TME) has emerged as a hot topic in tumor therapy and the TME determines the effectiveness of immunotherapy (Noguchi et al., 2017; Wei et al., 2019; Wu et al., 2021). Immune checkpoint inhibitors have exhibited unprecedented responses in several cancer patients. However, only a subset of patients with a few neoplasm types benefits from these therapies such as non-small cell lung cancer (NSCLC) (Rizvi et al., 2015), melanoma (Eroglu et al., 2018) and kidney cancer. And most patients belong to high-level mutational burden and microsatellite instability subtypes (Diaz et al., 2015). However, the serious adverse effects of current immunotherapeutic approaches cannot be ignored. The absence of early diagnosis and preventive measures also contributes to the persistently high incidence of cancer. Hence, there is a great demand to explore novel molecular prognostic biomarkers and prospective therapeutic intervention targets for cancer.

Long non-coding RNA (LncRNA) is a class of highly versatile transcripts without protein-coding functions, which was initially considered as “noise” of genomic transcription without biological functions. But as the research continues to advance, lncRNA has sparked a wave of new research in diverse fields in recent years (Wilusz et al., 2009; Grote and Boon, 2018). LncRNA is implicated in numerous vital regulatory functions including cell cycle regulation, genomic imprinting, gene reprogramming, chromatin modification, transcriptional activation, and intranuclear transport, as well as the development and prevention of human diseases (Wang et al., 2011; Iyer et al., 2015). It has been demonstrated in many studies that lncRNA expression disorders are associated with a variety of cancers and these frequent aberrant expressions are closely related to tumorigenesis, development and metastasis (Ji et al., 2021; Feng et al., 2022). Related researches also find that lncRNA regulates drug resistance in multiple tumor types on top of its oncogenic function (Barik et al., 2021; Zhou L. et al., 2022; Zhou Y. et al., 2022). Taking into account the critical functions of lncRNA in carcinoma, the identification of specific lncRNA in carcinoma and the development of lncRNA-based therapeutic strategies will be of great interest. More recently, based on the analysis of transcriptome data from public databases (Lu et al., 2021), the researchers identified PRR7-AS1 as one of a series of metabolism-related lncRNA in colorectal cancer (CRC). And PRR7-AS1 has also been described to act as a prognostic biomarker for patients with hepatocellular carcinoma (HCC) and is linked to immune cell infiltration (Lu et al., 2022). Non-etheless, the studies performed on PRR7-AS1 were not exhaustive and in-depth analysis in pan-cancer is absent.

In the present study, we performed a comprehensive and holistic bioinformatic analysis of PRR7-AS1 in pan-cancer using multiple public databases to systematically illustrate the profile of PRR7-AS1, including expression levels, prognosis and immune value. Our study facilitates insights into the pivotal roles of PRR7-AS1 in tumorigenesis and tumor immunity, and furnishes a useful reference for PRR7-AS1-based anti-tumor therapeutic targets.

## Materials and methods

### The UALCAN database analysis

UALCAN database is an open standards-based platform containing RNA-seq and clinical information from patients with 31 cancer types, which provides in-depth analysis of gene expression status between tumor and paired normal tissues (Chandrashekar et al., 2022). Herein, we obtained the results of pan-cancer observation of PRR7-AS1 expression in tumor versus normal tissues from the module of “TCGA.”

### Sangerbox 3.0 database analysis

The Sangerbox 3.0 is the most comprehensive online platform for exploring the effects of bioinformatics on human cancer (Shen W. et al., 2022). On the foundation of The Cancer Genome Atlas (TCGA) and Genotype-Tissue Expression (GTEx) datasets, the expression differences and prognosis values of PRR7-AS1 in diverse cancers versus adjacent normal tissues were analyzed by using the “Pan-cancer analysis tool.” Log-rank were used to construct the Kaplan-Meier plots. And the cutoff value of each continuous variable was decided by the optimal value. All abbreviations are summarized in Supplementary Table S1. In addition, the association between PRR7-AS1 expression status and the degree of immune cell infiltration and genomic heterogeneity was examined separately by Spearman’s correlation method. The ESTIMATE algorithm was used to acquire the relevance of PRR7-AS1 expression levels with stromal, immune, and ESTIMATE scores for each patient in diverse tumors. The data was log2 (x + 0.001) transformed. Furthermore, we conducted functional enrichment analysis of the protein-coding genes identified by PRR7-AS1 using the module of “GO and KEGG analysis tool.” FDR was further calculated using Benjamini & Hochberg methods. The top ten items for each enrichment analysis were generated and displayed by Sangerbox. The *p*-value under 0.05 was considered for statistical significance.

### ASSISTANT for clinical bioinformatics database analysis

ACLBI (https://www.aclbi.com/) is a comprehensive multi-dataset integrated online bioinformatics analysis platform. The expression levels of PRR7-AS1 across all tumors were investigated *via* the ACLBI database from The Cancer Genome Atlas (TCGA) datasets. Additionally, we obtained the relevance of PRR7-AS1 expression status to multiple immune infiltrating cells by different computational methods including XCELL, TIMER and MCPCOUNTER using the “Pan-Cancer Analysis” module. We further generated Spearman correlation analysis scatter plots of immune checkpoint-associated gene and PRR7-AS1 gene expression in multiple cancers using the TCGA module. Significance was considered at values of *p* < 0.05.

### GEPIA2 database analysis

Herein, the “Survival Plot” module in GEPIA2 was used to measure the connection between PRR7-AS1 expression and prognosis (OS, RFS) of cancers (Tang et al., 2019). Classification of the samples into high and low groups depending upon the median of the expression values. Furthermore, the module of “Stage Plot” was used to probe the discrepancy of PRR7-AS1 expression at different stages. Log2 (TPM+1) was used to transform expression data from the violin plot. A *p*-value under 0.05 was considered for statistical significance.

### Lnc2Cancer 3.0 database analysis

The Lnc2Cancer 3.0 database is a precious platform for illuminating the relationship between lncRNA and cancer (Gao et al., 2021). The expression levels of PRR7-AS1 across all tumors were investigated by the “box plot” module based on the TCGA datasets. Under the “Stage Plotting” module, the results of the PRR7-AS1 expression in distinct pathological stages of neoplasms were presented in the form of violin plots. Additionally, we chose the “Survival” module to assess the relevance between PRR7-AS1 expression status and disease-free survival (DFS), and overall survival (OS) by the log-rank approach. The samples were separated into two groups in accordance with the median gene expression level in all patients. The significance of the difference was determined at *p*-value <0.05.

### The ENCORI database analysis

The ENCORI is a platform developed for experimentally validated miRNA target interactions based on CLIP sequencing and degradome sequencing data, in addition to providing visualization and analysis of large-scale datasets (Li et al., 2014). The module of “Pan-Cancer” was employed to obtain the expression profile of PRR7-AS1 between tumors and their paired para-cancerous normal tissues. Significant differences were implied when *p*-values were <0.05. Moreover, the miRNA-Target module was used to predict lncRNA-targeted miRNAs and miRNA-targeted mRNAs.

### The lnCAR database analysis

LnCAR (https://lncar.renlab.org/) is a comprehensive interactive tool for cancer-related microarray data, which re-annotates public microarray expression data from more than 57,000 different cancer samples. The co-expression network and KEGG pathway of PRR7-AS1 in liver cancer tissues were analyzed in the LnCAR database. The significance criteria for filtering were set at *p* < 0.05.

### The TIDE database analysis

Tumor Immune Dysfunction and Exclusion (TIDE) (Fu et al., 2020) is a composite score of tumor immune dysfunction and immune escape, which was used to assess the clinical effectiveness of immunosuppressive therapy. We explored the performance of PRR7-AS1 in immune checkpoint blockade treatment using the “Biomarker Evaluation” modules based on the ability of known biomarkers to predict response outcomes and overall survival. In addition, we used the TIDE algorithm to compute PRR7-AS1 gene signatures for T cell dysfunction, which were calculated from a large cancer clinical dataset.

### Cell culture

The human colorectal cancer cell HCT116 and DLD1 were purchased from cell bank of the Chinese Academy of Sciences (Shanghai, China). HCT116 and DLD1 cells were maintained in McCoy’s 5A medium (GNM16600, GENOM) and RPMI 1640 medium (GENOM, GNM31800) respectively. Both of them were cultured at 37°C under 5% CO_2_. Cell culture dishes/plates and centrifuge tubes were purchased from NEST Biotechnology Co., Ltd.

### Drug treatment

JQ1 (S7110) and I-BET-762 (S7189) were purchased from Selleck (Shanghai, China). 2 × 105 cells were seeded in 6-well plates and added JQ1-1uM or I-BET-762 2 µM for 24 h.

### RNA isolation and quantitative PCR

The isolation of RNA was conducted with Trizol (Qiagen, 1023537) reagent. The qPCR assays were carried out as previously described (Li et al., 2018). β-actin was used as the endogenous control. The primer sequences used in this study are shown below:

PRR7-AS1-F: AAT​GGT​GGC​TAG​GAA​CAC​GG, PRR7-AS1-R: AGC​TCA​GTC​TGG​TAG​GGA​GG; ACTIN-F: CAC​CAA​CTG​GGA​CGA​CAT, ACTIN-R: ACA​GCC​TGG​ATA​GCA​ACG.

### Statistical analysis

Data analysis was performed using the two-sided Student’s t-test by GraphPad Prism 7 software. Data represent the mean ± SD, n ≥ 3. *p*-value <0.05 was considered as statistically significant.

## Results

### PRR7-AS1 expression analysis in pan-cancer

**FIGURE 1:**
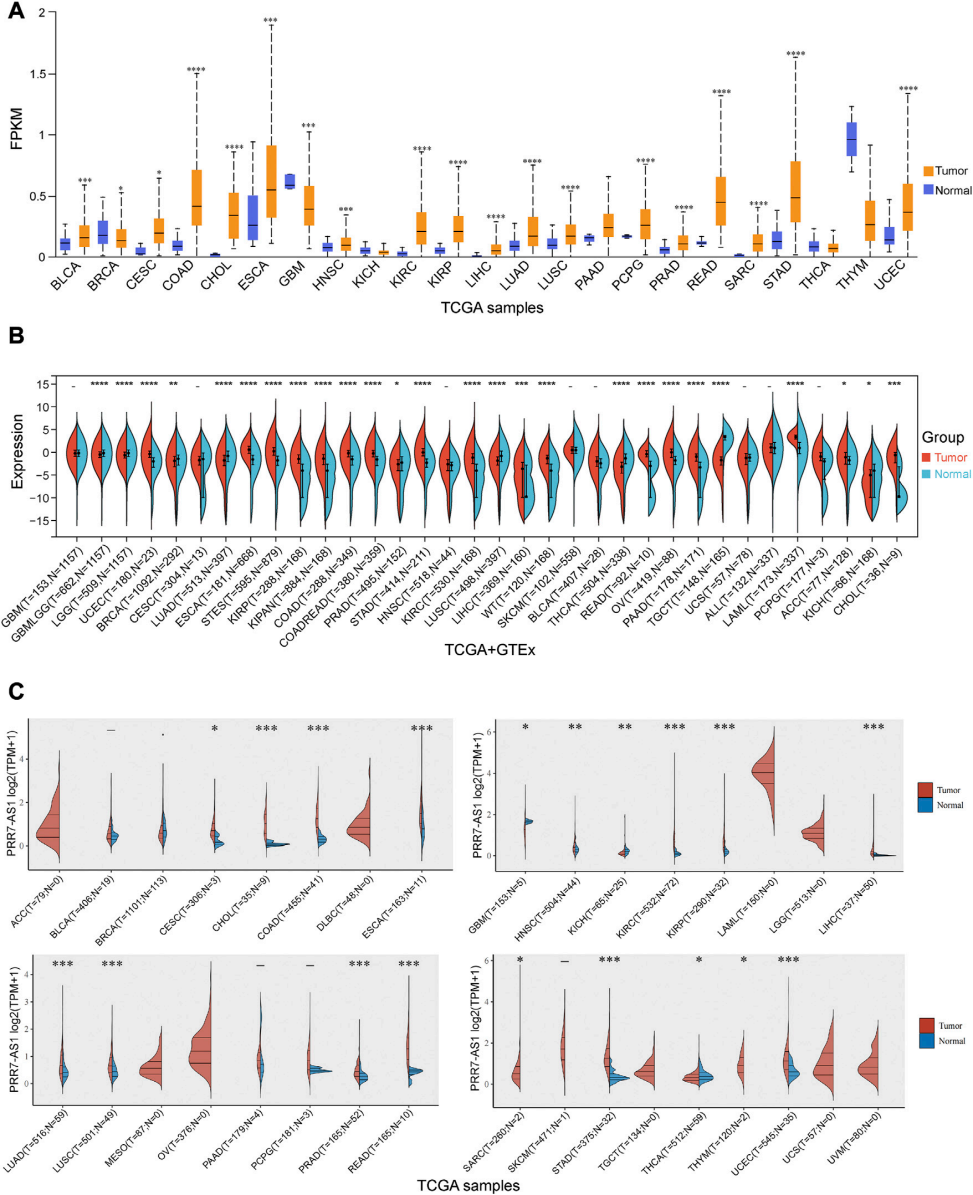
Expression levels of PRR7-AS1 in pan-cancer. **(A)** The expression levels of PRR7-AS1 in normal and tumor tissues were analyzed using the UALCAN database. **(B)** The expression differences of PRR7-AS1 between normal and corresponding tumor tissues from the Sangerbox database. **(C)** Differential expression levels of PRR7-AS1 in tumor and normal tissues were analyzed using the ACLBI database. *p < 0.05, **p < 0.01, ***p < 0.001, ****p < 0.0001.

To derive an overall profile of PRR7-AS1 expression status in cancer and paired normal tissues, we performed an analysis of PRR7-AS1 gene expression levels by using UALCAN database (Figure 1A). The outcomes revealed that the expression of PRR7-AS1 exhibited inconsistent levels in different types of cancers. In addition, Supplementary Figure S1A illustrated that PRR7-AS1 was ubiquitously expressed in a diverse range of normal human tissues such as immune, visceral, muscle, endocrine and reproductive tissues. At the cellular level, the CCLE dataset in the LncSEA database (Chen et al., 2021) indicated that PRR7-AS1 was maximally upregulated in melanoma versus other tumor types (Supplementary Figure S1B). In order to further acquire more reliable results and make the performance more representative, we proceeded to analyze the expression levels of PRR7-AS1 in pan-cancer by four additional databases-Sangerbox 3.0, ACLBI, Lnc2Cancer 3.0 and ENCORI (Figures 1B, C and Supplementary Figure S2). Consequently, we chose eight candidate tumors that had markedly elevated PRR7-AS1 expression in all of the above-mentioned databases. The eight tumor types contained CHOL, COAD, ESCA, KIRC, KIRP, LIHC, STAD, and UCEC. In addition, an assessment of the connection between PRR7-AS1 gene expression status and the pathological stage of cancer patients in different tumor types showed that PRR7-AS1 expression levels were remarkably correlated with tumor stage in certain cancer types (Supplementary Figure S3). Hence, PRR7-AS1 can be taken as a candidate diagnostic biomarker for some human cancer types.

### Multifaceted prognostic value of PRR7-AS1 in pan-cancer

**FIGURE 2:**
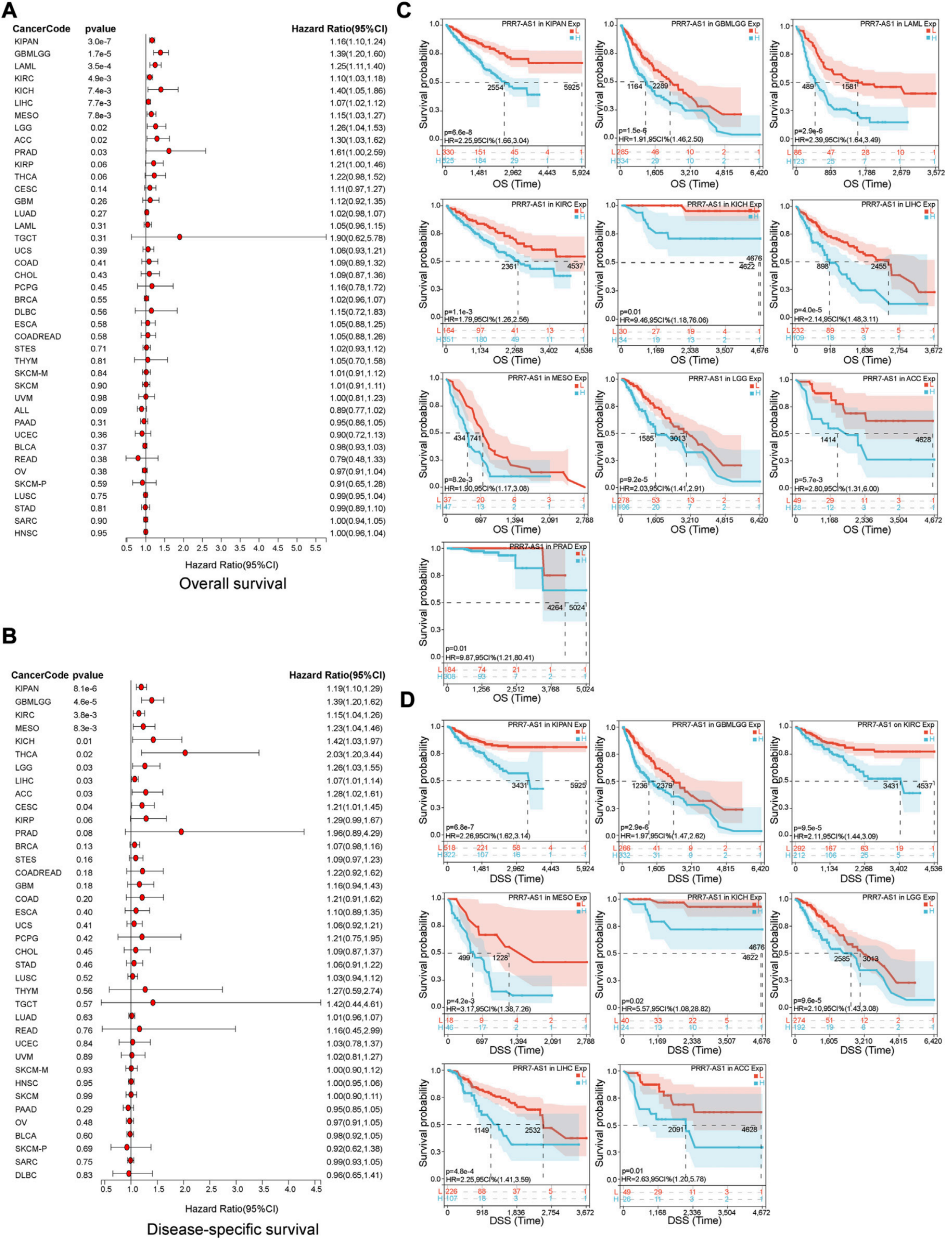
Prognostic value of PRR7-AS1 for overall survival (OS) and disease-specific survival (DSS) in pan-cancer. The forest plots showing the results of Cox regression analysis for **(A)** OS and **(B)** DSS. Kaplan-Meier analysis of **(C)** OS and **(D)** DSS for PRR7-AS1 in different tumors.

**FIGURE 3:**
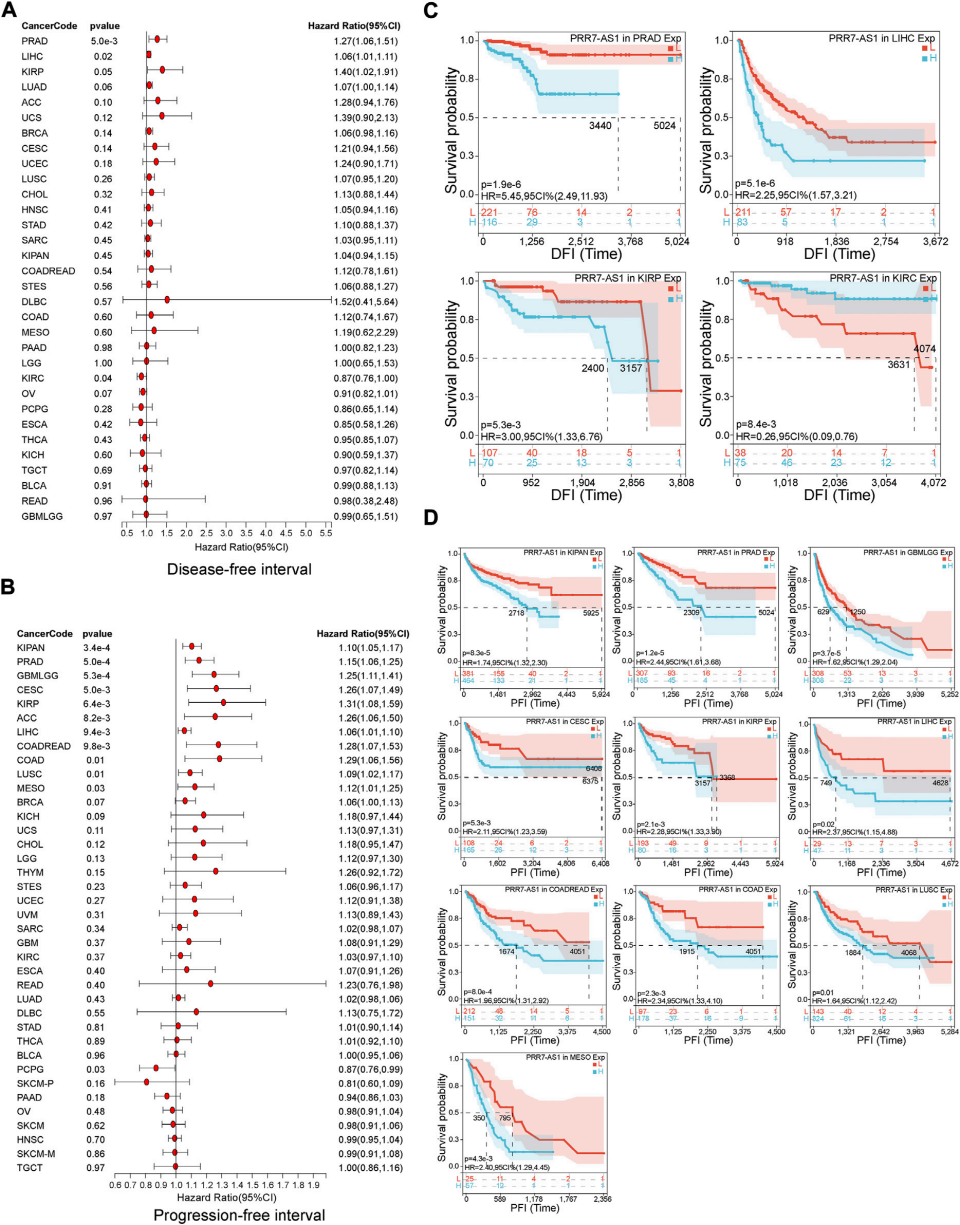
Prognostic value of PRR7-AS1 for disease-free survival (DFI) and progression-free interval (PFI) in pan-cancer. The forest plots showing the results of Cox regression analysis for **(A)** DFI and **(B)** PFI. Kaplan-Meier analysis of **(C)** DFI and **(D)** PFI for PRR7-AS1 in different tumors.

The value of PRR7-AS1 expression status in predicting the prognosis of tumor sufferers was analyzed by the TCGA and GTEx cohorts. A Cox proportional hazards regression model was developed to investigate the potential of PRR7-AS1 as a prognostic factor for each tumor type using the Sangerbox database. We combined PRR7-AS1 expression levels with multiple indicators related to the survival of cancer patients, including overall survival (OS), disease-specific survival (DSS), disease-free survival (DFI) and progression-free interval (PFI), and presented the results as forest plots (Figures 2A, B; Figures 3A, B). Furthermore, we evaluated the effect of PRR7-AS1 on cancer prognosis by utilizing Kaplan-Meier (KM) survival curves (Figures 2C, D; Figures 3C, D). Based on the results obtained from Sangerbox, high levels of PRR7-AS1 were connected with worse OS and DSS in ACC, KIRP, LIHC, and PRAD, and with worse OS/DSS/PFI in GBMLGG, LIHC, MESO, and KIPAN. Furthermore, in KIRC, KIRP, LIHC and PRAD, the expression status of PRR7-AS1 was related to worse DFI, while in CESC, COAD, KIRP, LUSC, PRAD, and COADREAD, elevated expression of PRR7-AS1 was connected with worse PFI. The prognostic values of PRR7-AS1 in tumors were further validated in GEPIA2 and Lnc2Cancer 3.0 database (Supplementary Figures S4A, B). We discovered that the high expression status of PRR7-AS1 in COAD, KICH, KIRC, KIRP, LGG, LIHC, MESO, and PRAD was related to poorer prognosis in the two databases. Taken together, these results demonstrated the potential values of PRR7-AS1 in the prognostic assessment for certain types of cancer patients. PRR7-AS1 is likely to be a candidate prognostic biomarker.

### TME analysis of PRR7-AS1 in pan-cancer

**FIGURE 4:**
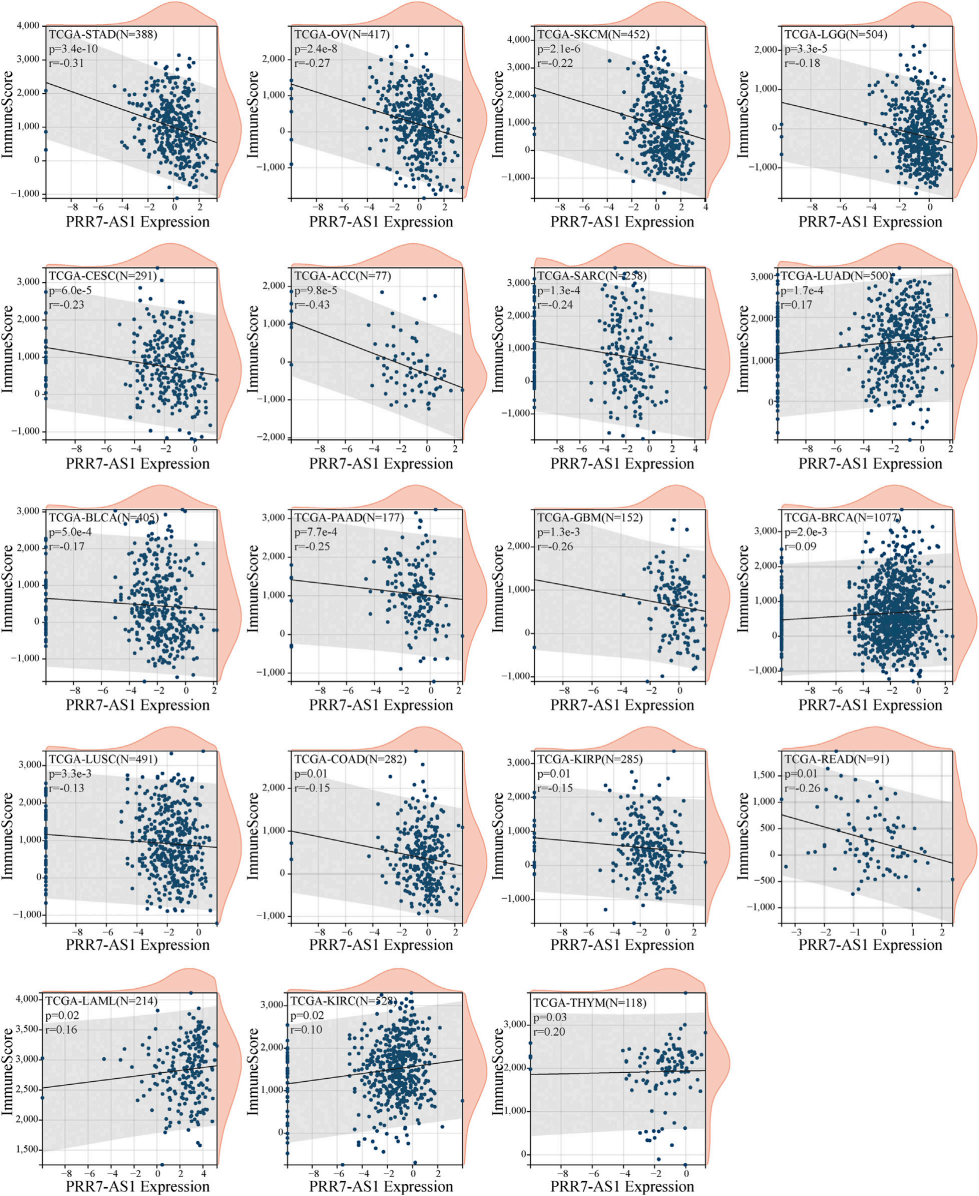
Correlation between PRR7-AS1 expression levels and immune score in pan-cancer. Correlation was tested by Spearman methods.

To begin, the correlation was investigated between PRR7-AS1 expression status and immune score (Figure 4). We detected that PRR7-AS1 expression levels were significantly connected with 19 of the 33 cancer types. Among them, a negative correlation was found in STAD, OV, SKCM, LGG, CESC, ACC, SARC, BLCA, PAAD, GBM, LUSC, COAD, KIRP, and READ. A positive correlation was found in LUAD, BRCA, LAML, KIRC, and THYM. Besides, the expression status of PRR7-AS1 in STAD, SARC, SKCM, LUSC, KIRP, BLCA, BRCA, LGG, PAAD, OV, HNSC, GBM, READ, LIHC, ACC, ESCA, UCS, and COAD was markedly negatively connected with stromal scores, while was positively correlated in LAML and TGCT (Supplementary Figure S5). The ESTIMATE Score was the total sum of the Stromal Score and the Immune Score, representing the overall ratio of these two components in TME. Similarly, the expression status of PRR7-AS1 was explored the correlation with the ESTIMATE Score in pan-cancer (Supplementary Figure S6). Taken together, we concluded that PRR7-AS1 expression was negatively linked to immune score, stromal score, and ESTIMATE score in 13 tumor types: ACC, BLCA, COAD, GBM, KIRP, LGG, LUSC, OV, PAAD, READ, SARC, SKCM, and STAD.

### Immune cell infiltration analysis of PRR7-AS1 in pan-cancer

**FIGURE 5:**
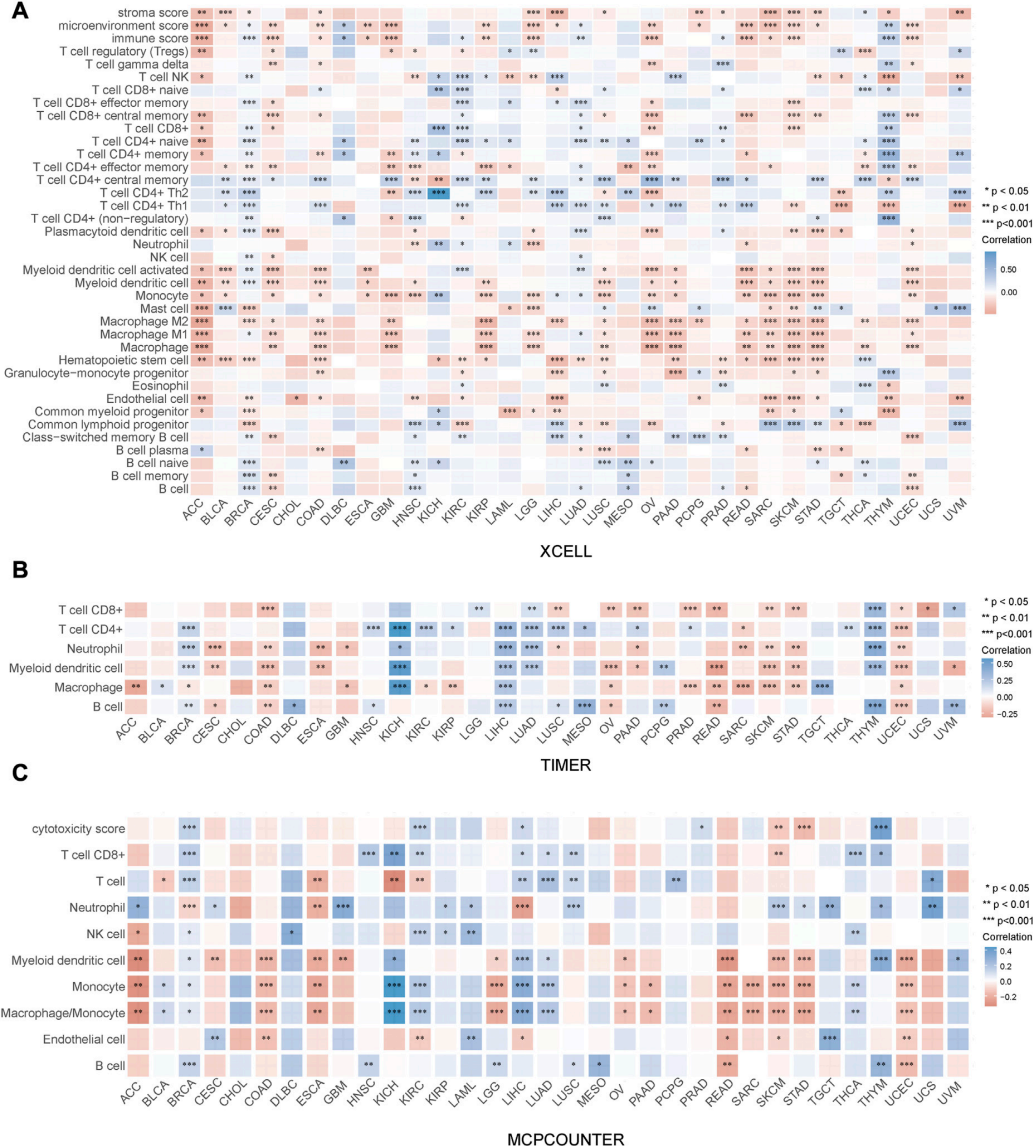
Correlation between PRR7-AS1 expression and immune cell infiltration. The expression of PRR7-AS1 was significantly correlated with various immune infiltrating cells based on the **(A)** XCELL, **(B)** TIMER and **(C)** MCPCOUNTER algorithm. Correlation was tested by Spearman methods. *p < 0.05, **p < 0.01, ***p < 0.001, ****p < 0.0001.

The immune status of the TME is determined by the reciprocal modulation of tumor cells and tumor-infiltrating immune cells (TIICs) (Liu et al., 2019). TIICs perform a pivotal function in the development of tumors. Therefore, we were interested in exploring the relation between PRR7-AS1 expression status and TIICs. Firstly, we examined the relation between PRR7-AS1 expression status and TIICs using ACLBI database and presented them as heat maps using three different algorithms- XCELL, TIMER and MCPCOUNTER, respectively (Figure 5). Afterwards, the connection between PRR7-AS1 expression and dendritic cells (DCs), CD8^+^ T, Macrophages, monocytes and Fibroblasts in the above 13 candidate tumors was investigated using the Sangerbox database (Supplementary Figures S7, S8). By the scatter diagram analysis, the expression levels of PRR7-AS1 were inversely linked to the majority of immune cells. Collectively, there was a remarkable negative connection between PRR7-AS1 expression and TIICs.

### Correlation analysis between PRR7-AS1 and immune checkpoint molecules expression

**FIGURE 6:**
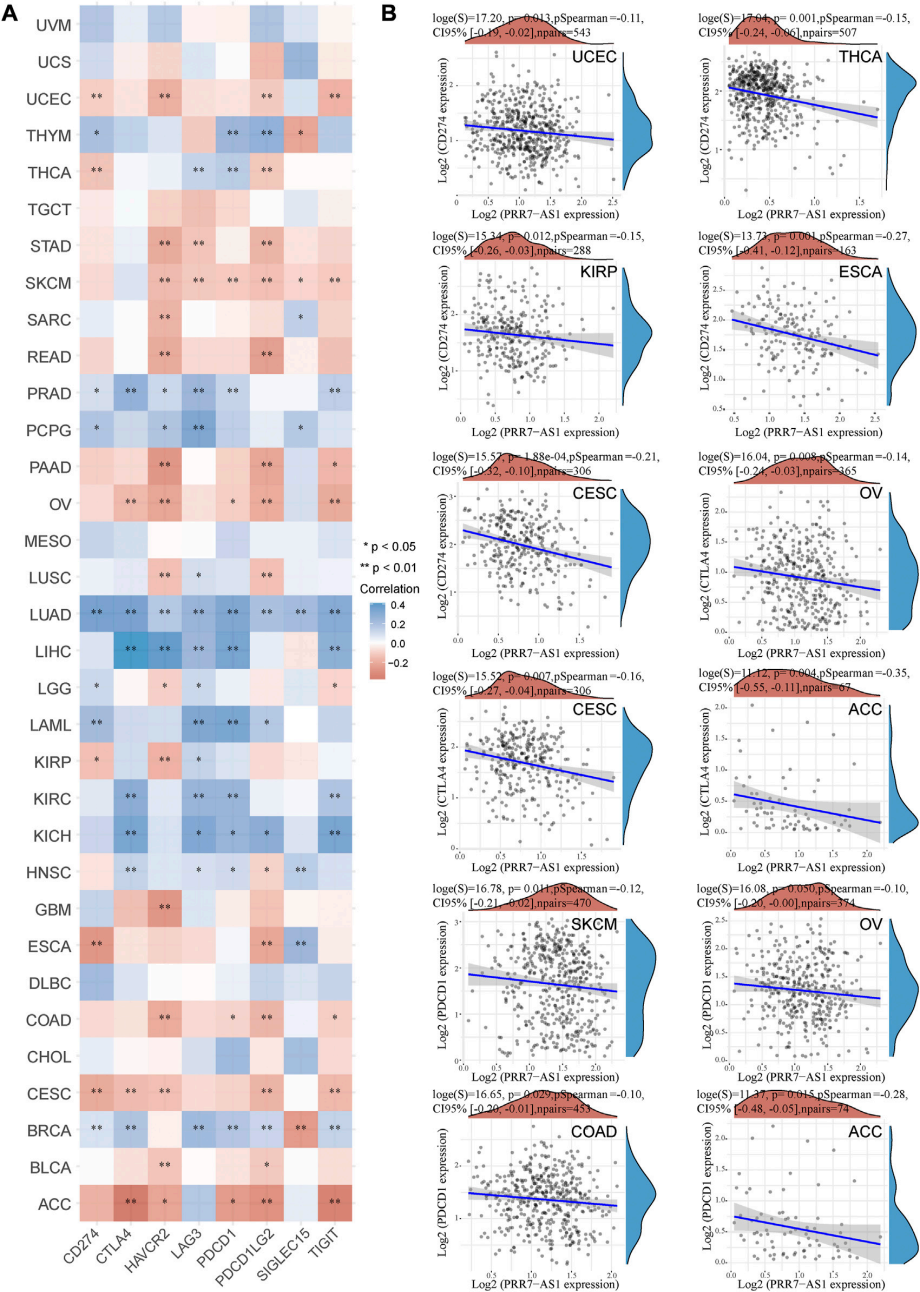
Correlation between PRR7-AS1 and immune checkpoint gene expression. **(A)** Heat map of PRR7-AS1 expression levels correlated with immune checkpoint genes in pan-cancer from the ACLBI database. **(B)** Scatter plots of PRR7-AS1 expression levels correlated with CD274, PDCD1 and CTLA4 in cancers. Correlation was tested by Spearman methods. *p < 0.05, **p < 0.01.

A crucial aspect of immune escape and immune checkpoint inhibitors (ICIs) treatment is the expression of immune checkpoint molecules (Hegde et al., 2016). Therefore, we examined the relation between PRR7-AS1 expression level and immune checkpoint molecules expression in different malignancy types utilizing the ACBLI database. As shown in Figure 6A, in ACC, CESC, COAD, OV, SKCM, and UCEC, increased PRR7-AS1 levels were inversely correlated with at least four immune checkpoint genes expression. While in a diverse range of cancers, such as BRCA, KICH, KIRC, LAML, LIHC, LUAD, PCPG, and PRAD, at an absolute minimum, four immune checkpoint genes were positively related to PRR7-AS1 expression status. Subsequently, we presented scatter plots showing a significant negative correlation between PRR7-AS1 and PD1, PDL1, and CTLA4 expression in different cancer types (Figure 6B). The above results suggested that PRR7-AS1 expression was strongly linked to immune checkpoint genes expression in the majority of cancer types.

### The connection between PRR7-AS1 expression and the efficacy of immunotherapy

**FIGURE 7:**
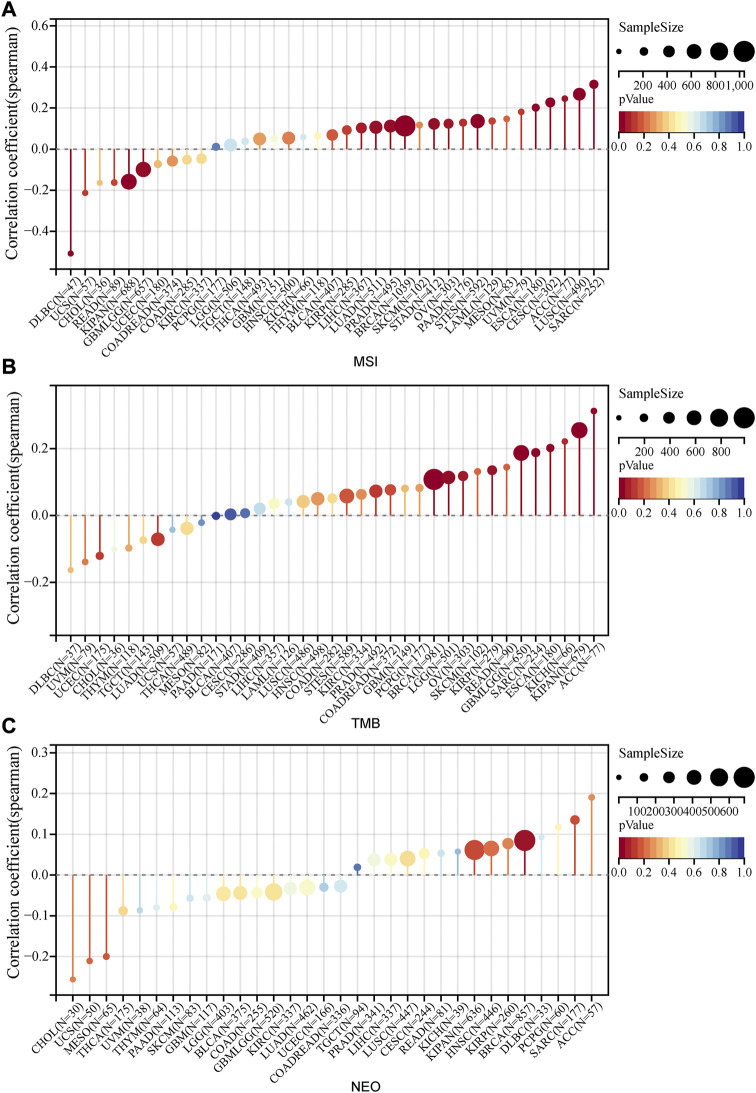
Lollipop plots of the correlation between PRR7-AS1 expression and MSI, TMB and NEO in pan-cancer. The correlation of PRR7-AS1 expression with **(A)** MSI, **(B)** TMB and **(C)** NEO. Correlation was tested by Spearman methods.

**FIGURE 8:**
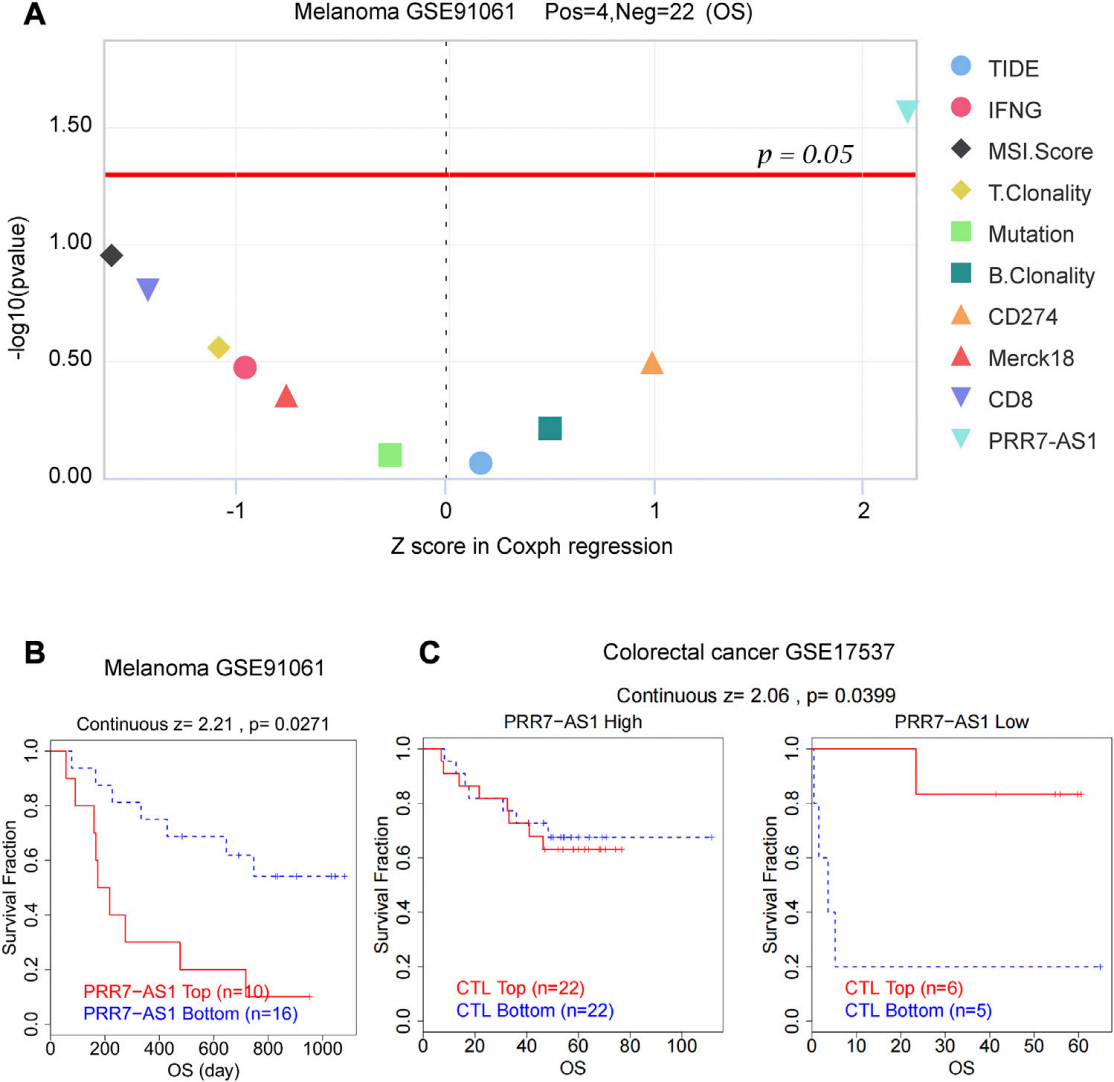
PRR7-AS1 predicted the efficacy of immunotherapy. **(A)** Z score in Coxph regression analysis of different predictors for immunotherapy efficacy in melanoma. **(B)** Kaplan-Meier curves of the OS in melanoma patients treated with PD1 inhibitors. **(C)** The overall survival analysis of patients in colorectal cancer with different PRR7-AS1 expression and CTL levels.

As important biomarkers of immunotherapy, TMB and MSI can predict the clinical efficacy of immunotherapy (Le et al., 2015; Samstein et al., 2019). Neoantigens (NEO) has also been shown to be a desirable target for T cell-based cancer immunotherapy (Zhang et al., 2021). Figure 7 displayed the correlation between PRR7-AS1 and MSI, TMB, and NEO in pan-cancer. PRR7-AS1 expression status was positively connected with MSI in SARC, LUSC, ACC, CESC, ESCA, STES, OV, STAD, BRCA, PRAD, and LUAD, while it was negatively related in KIPAN, GBMLGG and DLBC. PRR7-AS1 expression status was positively connected with MSI in SARC, LUSC, ACC, CESC, ESCA, STES, OV, STAD, BRCA, PRAD, and LUAD, while it was negatively related in KIPAN, GBMLGG, and DLBC. And in the association analysis of PRR7-AS1 expression with TMB, PRR7-AS1 showed a positive correlation in ACC, KIPAN, ESCA, SARC, CBMLGG, KIRP, OV, LGG, and BRCA. The PRR7-AS1 expression level was positively correlated with NEO in BRCA. Moreover, it was shown in Figure 8A that PRR7-AS1 was available to independently predict the response to immunotherapy. And PRR7-AS1 had the best predictive power for immune checkpoint blockade (ICB) efficacy compared to other well-recognized biomarkers. Patients with low PRR7-AS1 expression in melanoma responded better to PD1 inhibitor therapy (Figure 8B). In addition, Figure 8C illustrated that cytotoxic T cells (CTL)-mediated antitumor responses were stronger and accompanied by better prognosis in CRC patients with low PRR7-AS1 expression, implying its potential as a possible new target for immunotherapy.

### Functional enrichment analysis of PRR7-AS1

**FIGURE 9:**
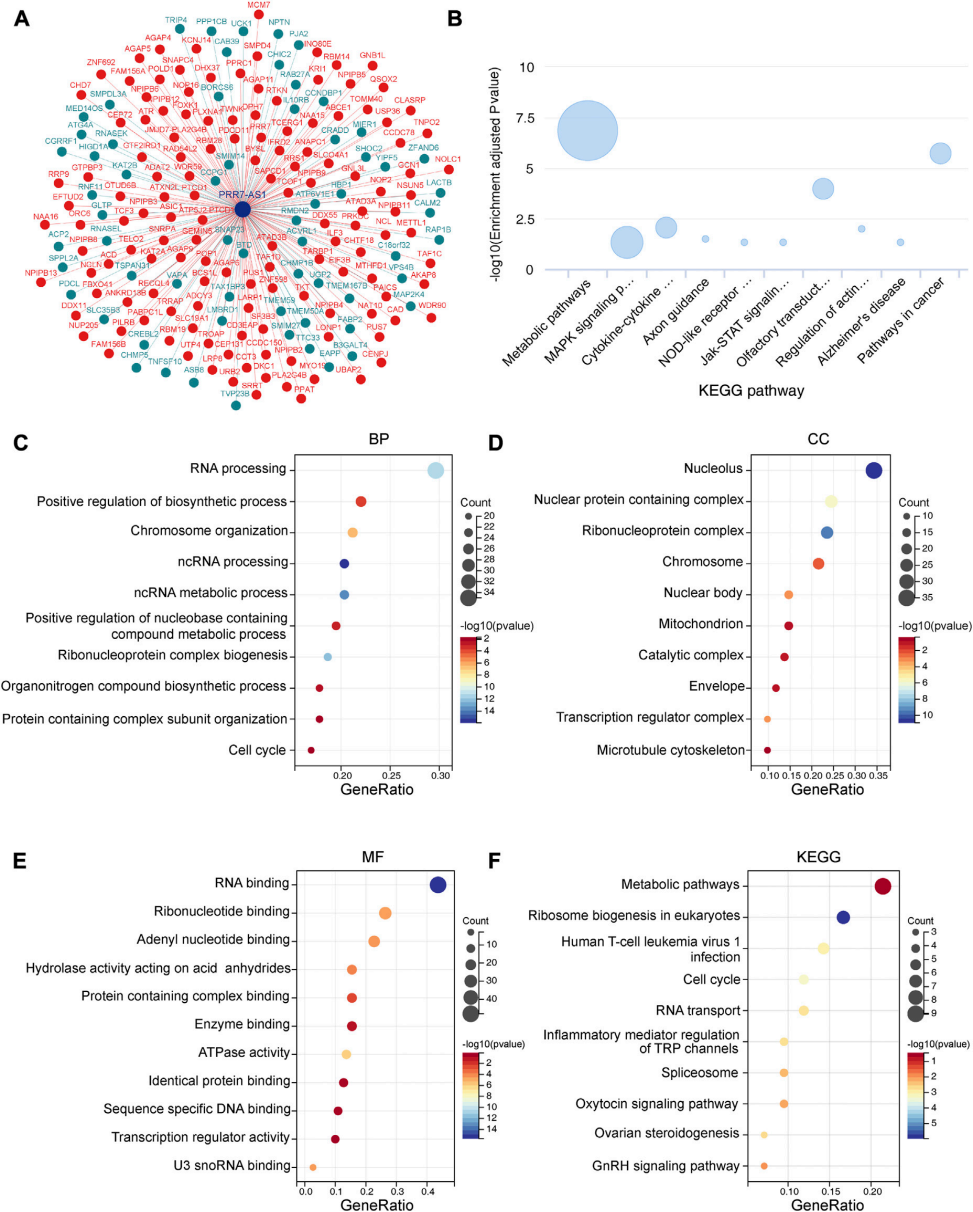
Functional enrichment analysis of PRR7-AS1. **(A)** The co-expression interaction network of PRR7-AS1 from lnCAR database. **(B)** The KEGG pathway analysis of PRR7-AS1 by lnCAR database (CR_S36). **(C–F)** GO and KEGG analysis for PRR7-AS1 associated genes. (BP, Biological process; CC, Cellular component; MF, Molecular function; KEGG, kyoto encyclopedia of genes and genomes).

To identify the functional correlation of coding genes that are potentially regulated by PRR7-AS1, we performed a functional enrichment analysis. Considering that the expression levels and prognostic values of PRR7-AS1 were relatively higher in COAD, we generated a co-expression network drawn by 63 genes negatively associated with the expression of PRR7-AS1 and 137 positively associated genes in COAD (Figure 9A). Bubble map of the KEGG pathway derived from the lnCAR database showed that PRR7-AS1-related genes were mainly enriched in metabolic pathways (Figure 9B). Genes that were co-expressed with PRR7-AS1 were extracted from the lnCAR database, and the functional enrichment analysis was carried out with the Sangerbox database (Figures 9C–F). The outcomes indicated that the biological process (BP) was mostly enriched in RNA processing. The cellular component (CC) was primarily nucleolus. For the molecular function (MF), genes were mostly engaged in RNA binding. The KEGG pathway analysis suggested that PRR7-AS1-related genes may be primarily involved in the metabolic pathways.

### Super enhancer activity was responsible for PRR7-AS1 expression

**FIGURE 10:**
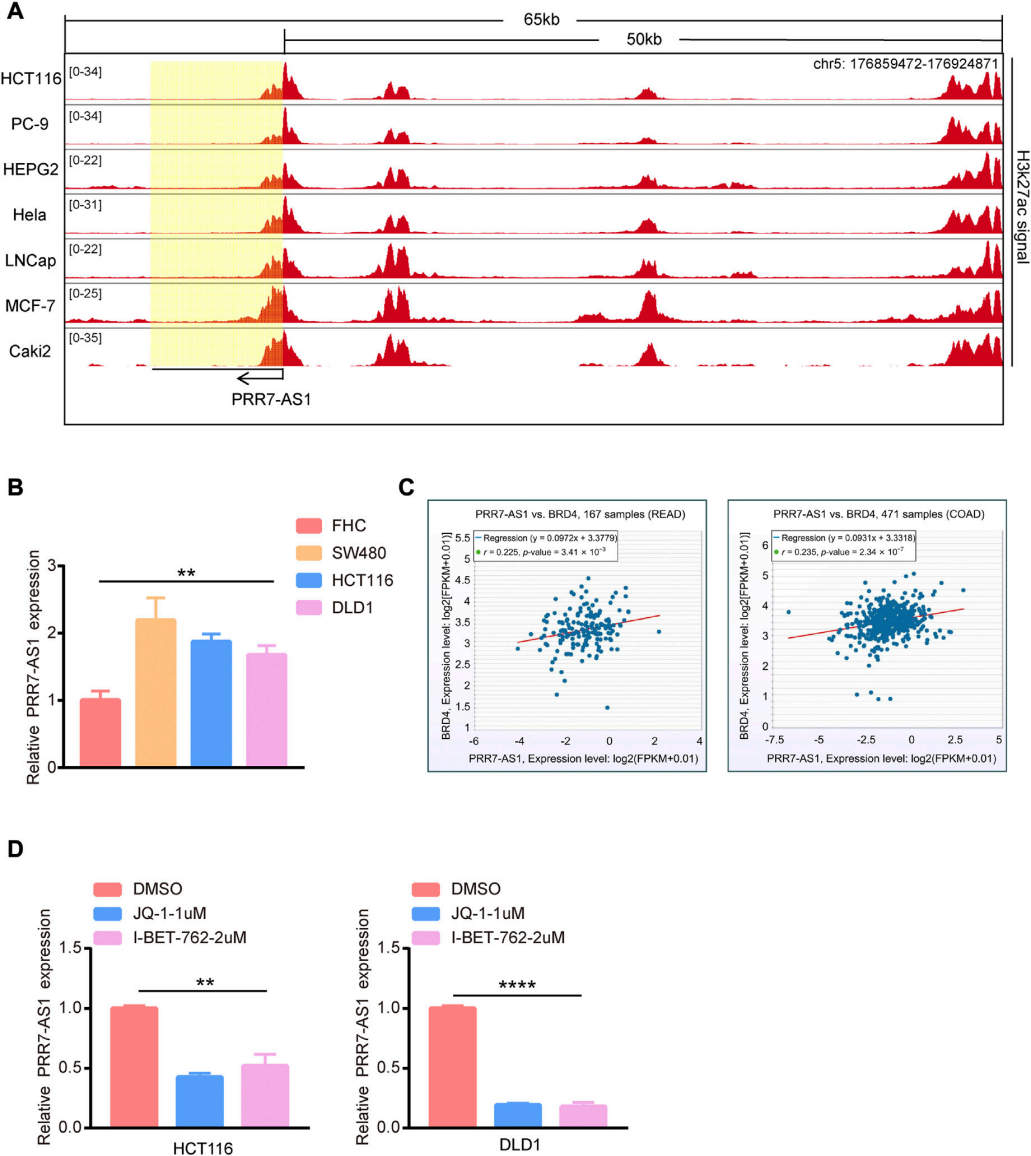
The effect of super enhancer activity on PRR7-AS1 expression. **(A)** H3K27ac signals in PRR7-AS1 gene loci in multiple cancer cells. The CHIP-seq data was downloaded from the ENCODE database. **(B)** The RNA expression levels of PRR7-AS1 were measured by qPCR in colorectal cancer cell lines. **(C)** The correlation between PRR7-AS1 and BRD4 expression in colorectal cancer was analyzed by using the ENCORI database. **(D)** The relative expression of PRR7-AS1 after JQ-1 and I-BET-762 treatment was detected by qPCR. **p < 0.01, ****p < 0.0001.

Now that PRR7-AS1 has key roles in tumorigenesis, we wondered the driving forces for its overexpression in tumors. We discovered abundant H3K27ac signals exiting PRR7-AS1 gene loci in multiple cancer cells by analyzing ChIP-seq data downloaded from the ENCODE database (Davis et al., 2018) (Figure 10A). Multiple clustered H3K27ac signals are the most used marker for super enhancers (Zhang et al., 2020). Hence, we supposed that PRR7-AS1 expression status was impacted by the super enhancer activity. In order to verify this hypothesis, we found PRR7-AS1 was also overexpressed in colorectal cancer cell lines compared with normal colon epithelial cell-FHC (Figure 10B). Importantly, in colorectal cancer, PRR7-AS1 expression was positively correlated with the expression of BRD4, the primary H3K27ac signal reader (Tasdemir et al., 2016) (Figure 10C). What’s more, treatment with BRD4 inhibitors (JQ-1 and I-BET-762) caused a significant reduction in the expression level of PRR7-AS1 (Figure 10D). Thus, we speculated that super enhancer activity might be participated in the regulation of PRR7-AS1 expression.

### Construction of PRR7-AS1 ceRNA network

**FIGURE 11:**
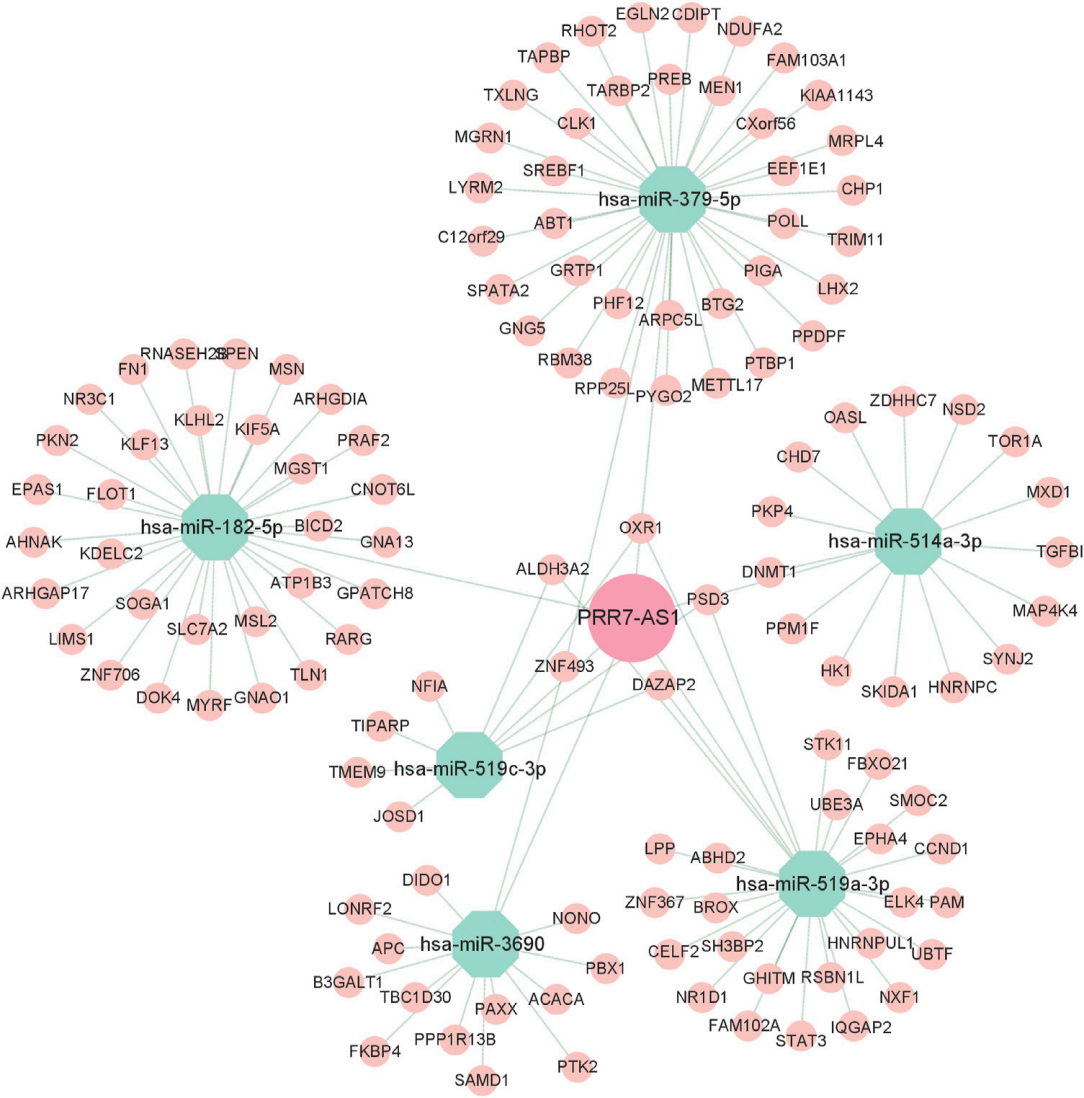
CeRNA regulatory networks for PRR7-AS1. Diamond represent lncRNA, Octagons represent miRNA, Circles represent mRNA.

To explore the regulatory mechanisms of PRR7-AS1, we used the ENCORI database to predict seven PRR7-AS1 targeted miRNAs. And the filter criteria were set to clip data ≥ 1, degradome data ≥0, pan-cancer ≥ 3. Interestingly, the expression levels of six of these miRNAs were negatively correlated with PRR7-AS1 in various tumors (Supplementary Figure S9). Subsequently, mRNAs that may share binding sites with the predicted seven miRNAs were screened in accordance with the following constraints: clip data ≥ 1, degradome data ≥ 1, pan-cancer ≥ 6, program Num ≥ 1. Therefore, a meaningful ceRNA network was obtained based on the relation between PRR7-AS1, miRNAs and mRNAs, which was visualized by applying Cytoscape (Saito et al., 2012) (Figure 11).

## Discussion

Although considerable advancements have been accomplished towards the diagnosis and treatment of tumors, new cases and deaths from tumors continue to rise annually (Sung et al., 2021). Therefore, searching for novel tumor biomarkers for predicting tumor behaviors and monitoring the response to treatment is of utmost importance. Still little data are known about lncRNA PRR7-AS1 in most tumor types. In this work, we focused on the analysis concerning the expression, prognosis and immune signatures of PRR7-AS1 in pan-cancer. To gain more knowledge about PRR7-AS1, we found high expression of PRR7-AS1 across four different databases in cancer, suggesting that PRR7-AS1 can be treated as a potential diagnostic biomarker in the majority of neoplasms. Simultaneously, we identified that high expression of PRR7-AS1 was relevant to the pathological stage of the neoplasms, which suggests that high expression of PRR7-AS1 contributes to the diagnosis of cancer sufferers in a clinical setting. Subsequently, our research showed that PRR7-AS1 could also act as an independent risk factor. Similarly, PRR7-AS1 could serve as a diagnostic and prognostic biomarker for HCC and contribute to the progression of the tumor microenvironment (TME) (Lu et al., 2022).

TME is utilized to assess the therapeutic efficacy of immunotherapy (Kessenbrock et al., 2010). TIICs are an integral component of the host immune response to tumors, which are inextricably intertwined with tumorigenesis, progression and metastasis (Steven and Seliger, 2018; Li et al., 2021). DC cells are antigen-presenting cells with a critical effect on the tumor-specific immune response (Melief, 2008). Macrophages also perform a vital function in cancer progression as sentinels of the immune system (Kuang et al., 2009). Extraordinarily, we identified an overall negative trend in the relation between PRR7-AS1 and immune infiltrating cells in most cancer types. Immunotherapy is performing an incrementally essential part in the management of cancer. Tumor cells tend to evade immune surveillance by modulating immune checkpoints (Havel et al., 2019). To tackle the challenge of immune escape, immunotherapies targeting immune checkpoints have garnered a considerable amount of attention. Particularly, the employment of CTLA4 and PD1/PD-L1 inhibitors has emerged as an important landmark in cancer immunotherapy (Zhang and Zhang, 2020). Notably, our outcomes detected that PRR7-AS1 expression status was inversely correlated with the immune checkpoint genes such as CD274, CTLA4, and PDCD1 in some carcinomas, which implies that cancer patients with high PRR7-AS1 expression status are probably not appropriate for the immune checkpoint inhibitors. TMB impacts the production potential of immunogenic peptides, thereby affecting the patient’s response to immunotherapy (Chan et al., 2019). TMB has been proven to correlate with the prognosis of immunotherapy for diverse cancer types (Samstein et al., 2019; Wang et al., 2019). Besides, MSI may also act as a potential biomarker to forecast immunotherapy effectiveness (Zhao et al., 2019). Hypermutation of cancer cells can produce neoantigens, which are an essential class of tumor high-specific antigens that can be locked by neoantigen-specific T-cell receptors. The immune activity of tumor neoantigens can be exploited to synthesize neoantigen vaccines to immunize patients, which can be therapeutically effective (Cohen et al., 2015; Tubb et al., 2018; Blass and Ott, 2021). Our results revealed that PRR7-AS1 expression was closely related to MSI, TMB and NEO in cancers, and influenced the immunotherapeutic response of patients, which provides new perspectives for improving treatment outcomes. Moreover, we found that CTL-mediated antitumor responses were influenced by PRR7-AS1 expression, suggesting that PRR7-AS1 may induce immune escape by impairing T-cell function. Thus, PRR7-AS1 might be a viable new target for immunotherapy.

Screening for unidentified lncRNA-interacting proteins for enrichment analysis is essential to understand the biological functions underlying lncRNA molecules. Our pathway enrichment analysis indicated that PRR7-AS1 probably contributed to the regulation of metabolic pathways. Recent study suggested that lncRNA PRR7-AS1 was identified as a metabolism-related lncRNA (Lu et al., 2021). Metabolic reprogramming is one of the representative features of tumors and targeting tumor metabolism has emerged as a hot topic (Hanahan and Weinberg, 2011; Vander Heiden and DeBerardinis, 2017). As researches continue to advance, lncRNAs also play pivotal roles in the metabolic changes of tumors (Lin, 2020). The aberrant expression of lncRNA may contribute to the dysregulation of key genes in glucose, lipid and amino acid metabolism (Muret et al., 2019; Zheng et al., 2019); (Shen Q. et al., 2022). Interestingly, metabolic reprogramming has a great impact on immunotherapy (Lv et al., 2022; Yang et al., 2022; Zhu et al., 2022). Therefore, now that PRR7-AS1 is closely correlated with immune microenvironment, further exploring the effect of PRR7-AS1 on tumor metabolism has profound clinical implications. Super enhancers (SEs) are large clusters of transcriptional enhancers that recruit much more intensive transcription factors and have more powerful transcriptional activation than typical enhancers (Hnisz et al., 2013; Jia et al., 2019). The majority of genes related to super enhancers are specific for determining cellular properties. Moreover, SEs have been proven to exert an essential role in cancer development (Chen et al., 2018), cell differentiation (Debek and Juszczynski, 2022) and immune response (Kim et al., 2021). In this study we found the super enhancer activity was responsible for PRR7-AS1 overexpression in tumors. Whether super enhancer-induced PRR7-AS1 could influence immune response by regulating metabolic process? This hypothesis warrants further validation through a series of *in vitro* and *in vivo* tests. Additionally, the ceRNA is thought to be a novel regulatory mechanism that acts through miRNA competition. MiRNAs modulate gene expression by binding with sequences in the 3′UTR of the target mRNA, causing target mRNA cleavage or translational repression (Garzon et al., 2010). In a complex ceRNA network, ceRNA genes are regulated by miRNAs while miRNAs could also interact with lncRNAs (Karreth and Pandolfi, 2013; Tay et al., 2014). In this study, we predicted PRR7-AS1-targeted miRNAs to construct a ceRNA network, providing a basis for further exploration of the regulatory mechanism of PRR7-AS1.

In summary, PRR7-AS1 expression was upregulated in most tumors and was closely associated with poor prognosis. PRR7-AS1 expression had a negative correlation with immune cell infiltration and immune checkpoint genes expression. PRR7-AS1 had a high predictive value for immunotherapy. PRR7-AS1 might be a predictive biomarker and a new target for immunotherapy. While numerous databases have provided a wealth of meaningful data for our analysis of the association of PRR7-AS1 with cancers. Our pan-cancer analysis of PRR7-AS1 still has several limitations. Our understanding of PRR7-AS1 is only the tip of the iceberg. However, more functions and mechanisms still need to be further investigated and elaborated in the clinical setting. And how PRR7-AS1 regulated the metabolic processes and the immune microenvironment? The causes and mechanisms of these problems still need to be further clarified.

## Conclusion

PRR7-AS1 had the potential to be a diagnostic, prognostic and immune biomarker for pan-cancer. PRR7-AS1 was correlated with an immunosuppressive microenvironment and was a new potential target for immunotherapy. Epigenetic factors were the driving forces for PRR7-AS1 overexpression in tumors.

## Data Availability

The original contributions presented in the study are included in the article/Supplementary Material, further inquiries can be directed to the corresponding authors.
