# Supplementary material for: Pan-cancer analysis of super enhancer-induced PRR7-AS1 as a potential prognostic and immunological biomarker
Source: Front Genet. 2023 Apr 6;14:1160599. doi: 10.3389/fgene.2023.1160599 (PMC10117660; doi:10.3389/fgene.2023.1160599)
Supplement: Supplementary file 1 [file DataSheet1.PDF]

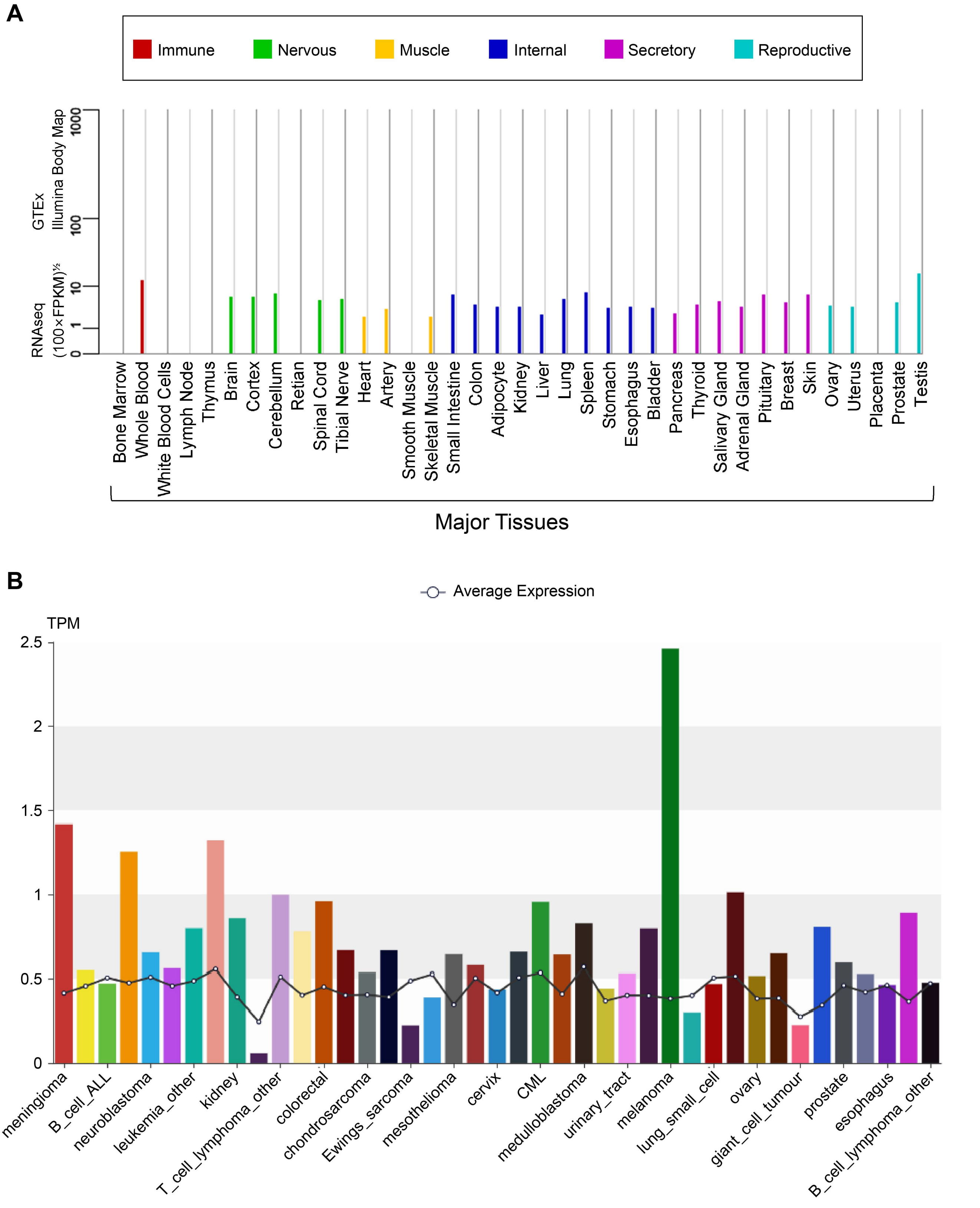

Supplementary Figure 1. Expression levels of PRR7-AS1 in pan-cancer. (A) Bar plots of PRR7-AS1 expression in diverse normal human tissues from TCGA and GTEx datasets. (B) Different expression levels of PRR7-AS1 in tumor cells from the CCLE dataset.

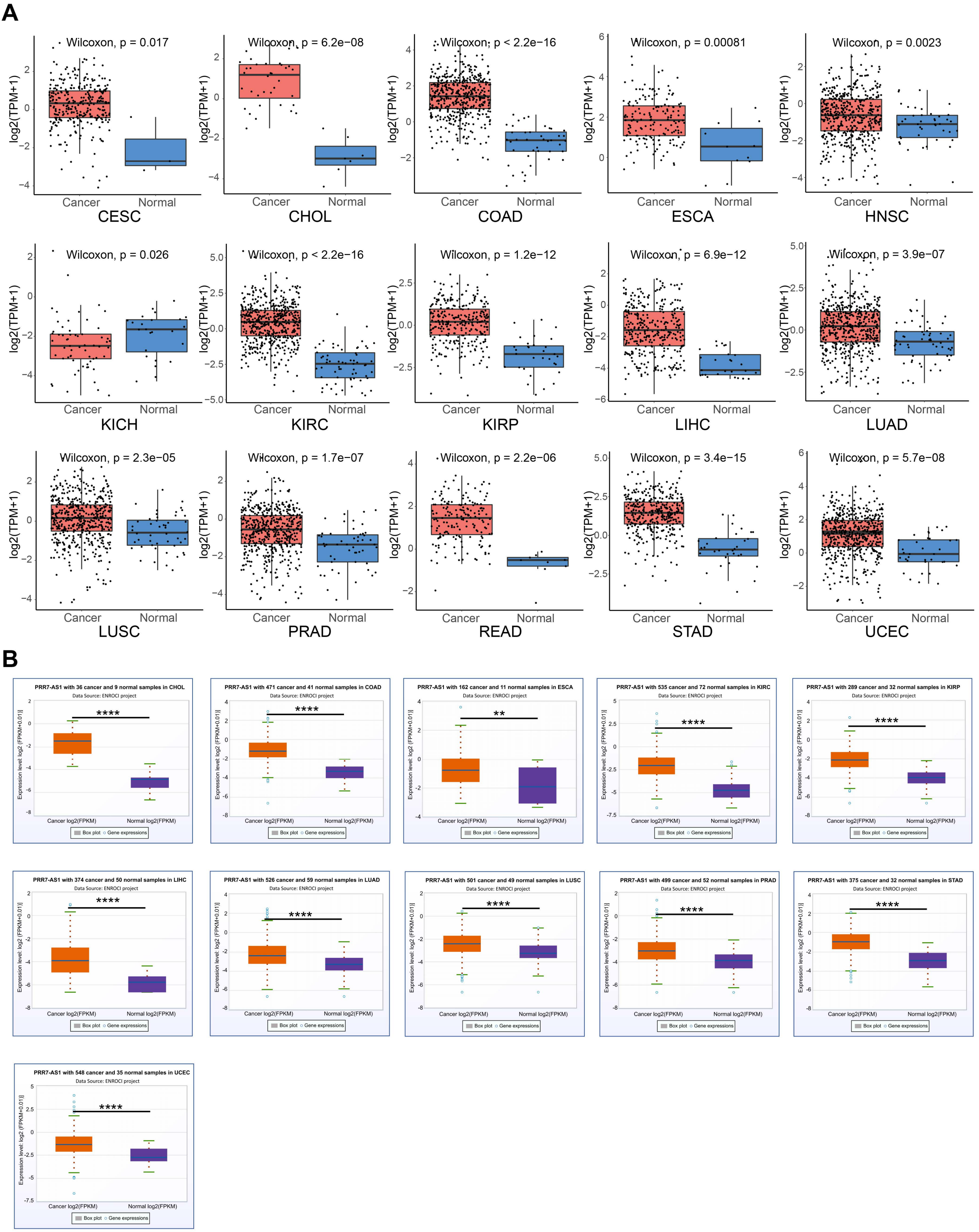

Supplementary Figure 2. Expression levels of PRR7-AS1 in pan-cancer. (A) PRR7-AS1 expression in different cancers using Lnc2Cancer 3.0 database. (B) The expression levels of PRR7-AS1 in cancers and paired normal tissues were analyzed via ENCORI database. *p < 0.05, **p < 0.01, ***p < 0.001, ****p < 0.0001.

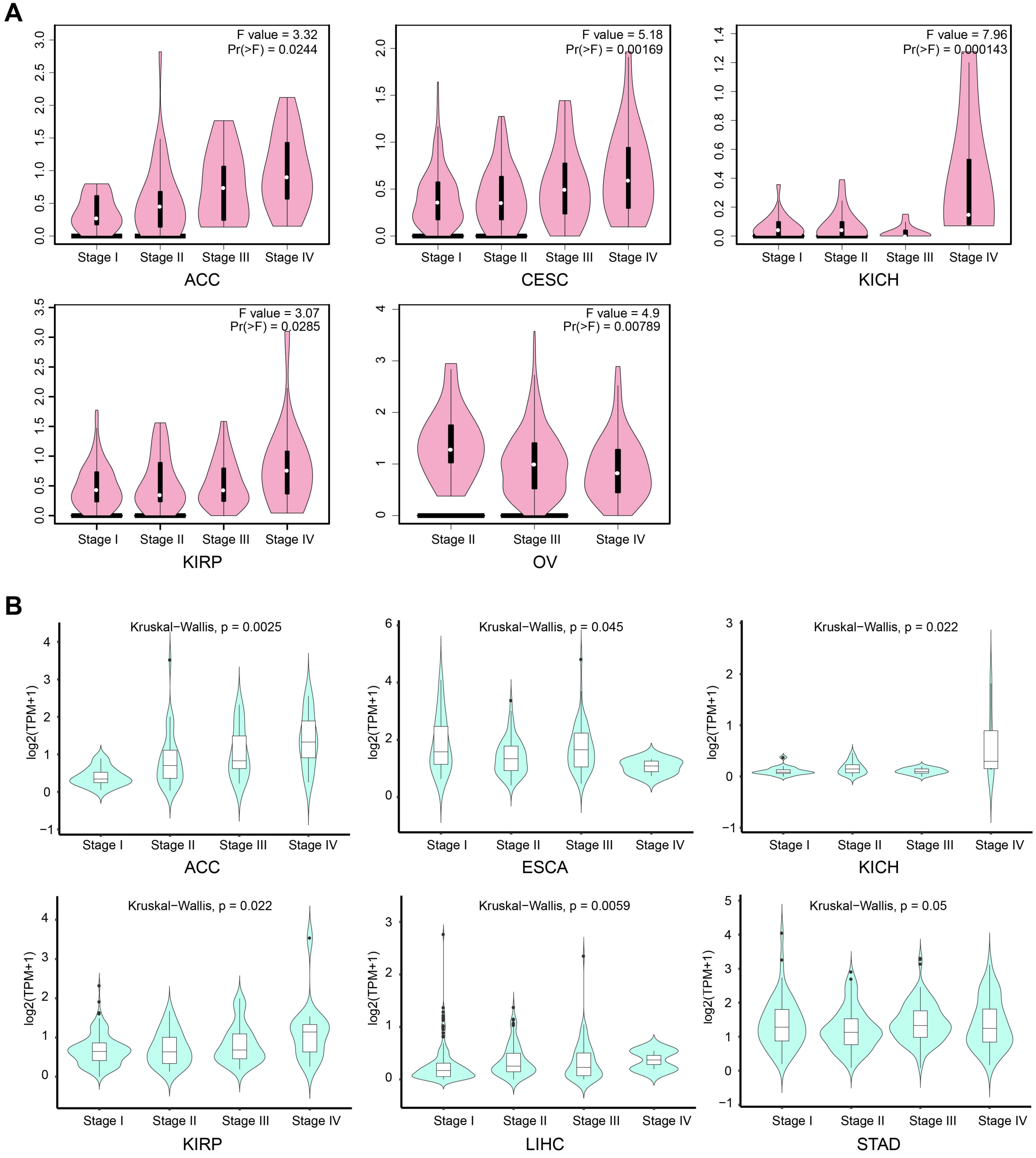

Supplementary Figure 3. The analysis of PRR7-AS1 expression levels at different pathological stages. Expression levels of PRR7-AS1 in different pathological stages in (A) GEPIA2 and (B) Lnc2Cancer 3.0 database.

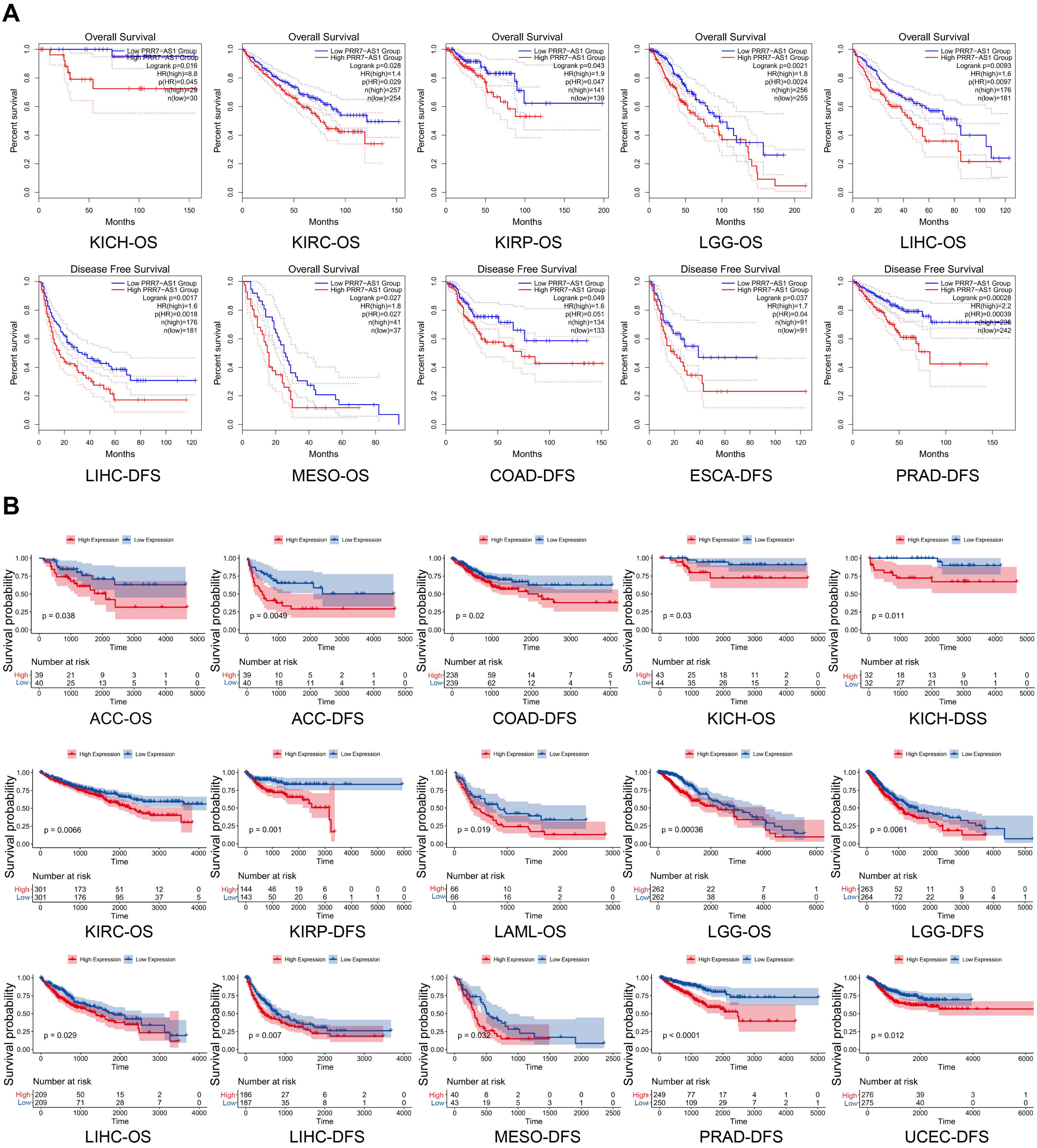

Supplementary Figure 4. Prognostic values of PRR7-AS1 in pan-cancer. (A) The OS and DSS of PRR7-AS1 in pan-cancer based on GEPIA2 database. (B) Survival analysis of PRR7-AS1 expression in pan-cancer by using Lnc2Cancer 3.0 database.

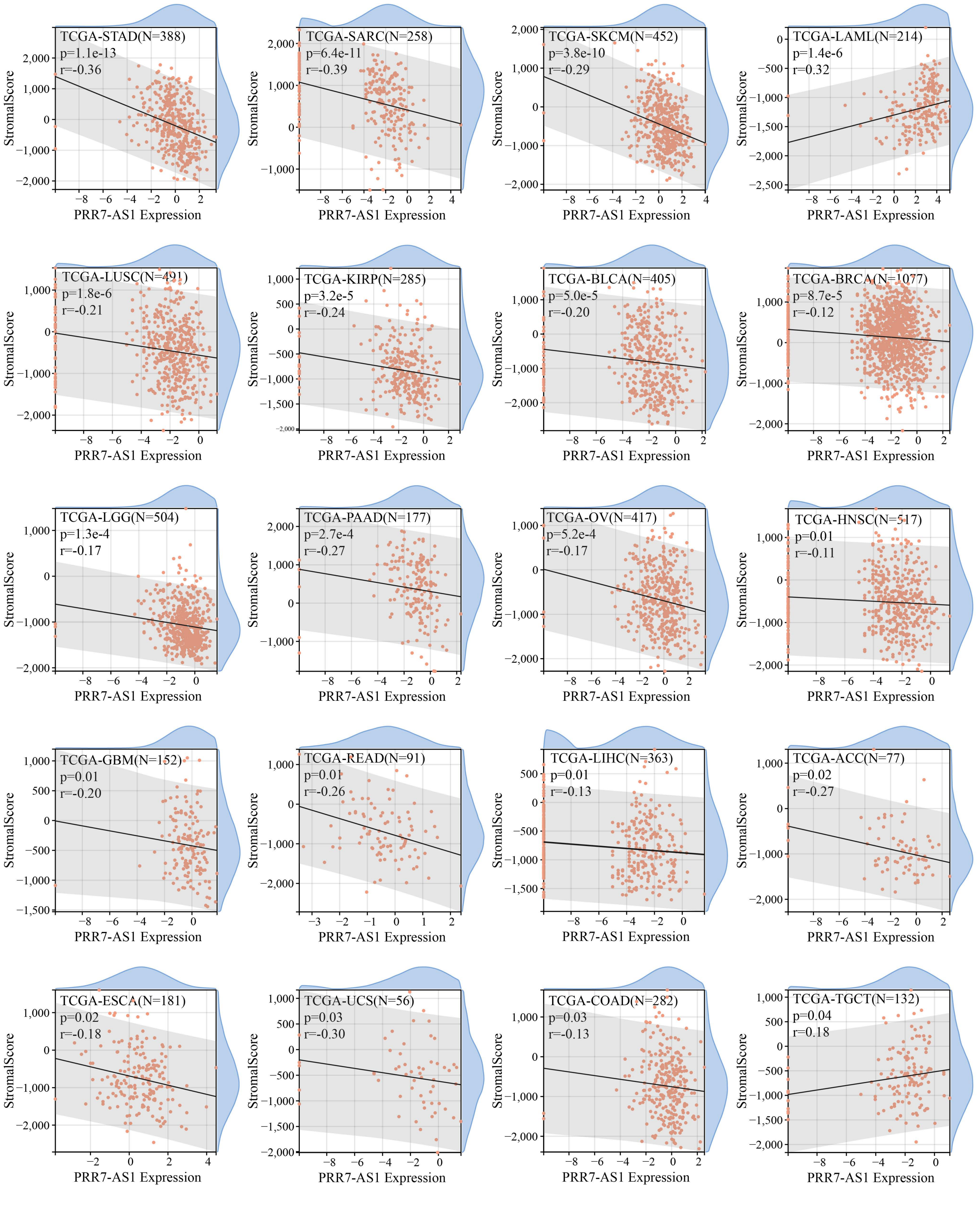

Supplementary Figure 5. Correlation between PRR7-AS1 expression levels and Stromal score in pan-cancer. Correlation was tested by Spearman methods.

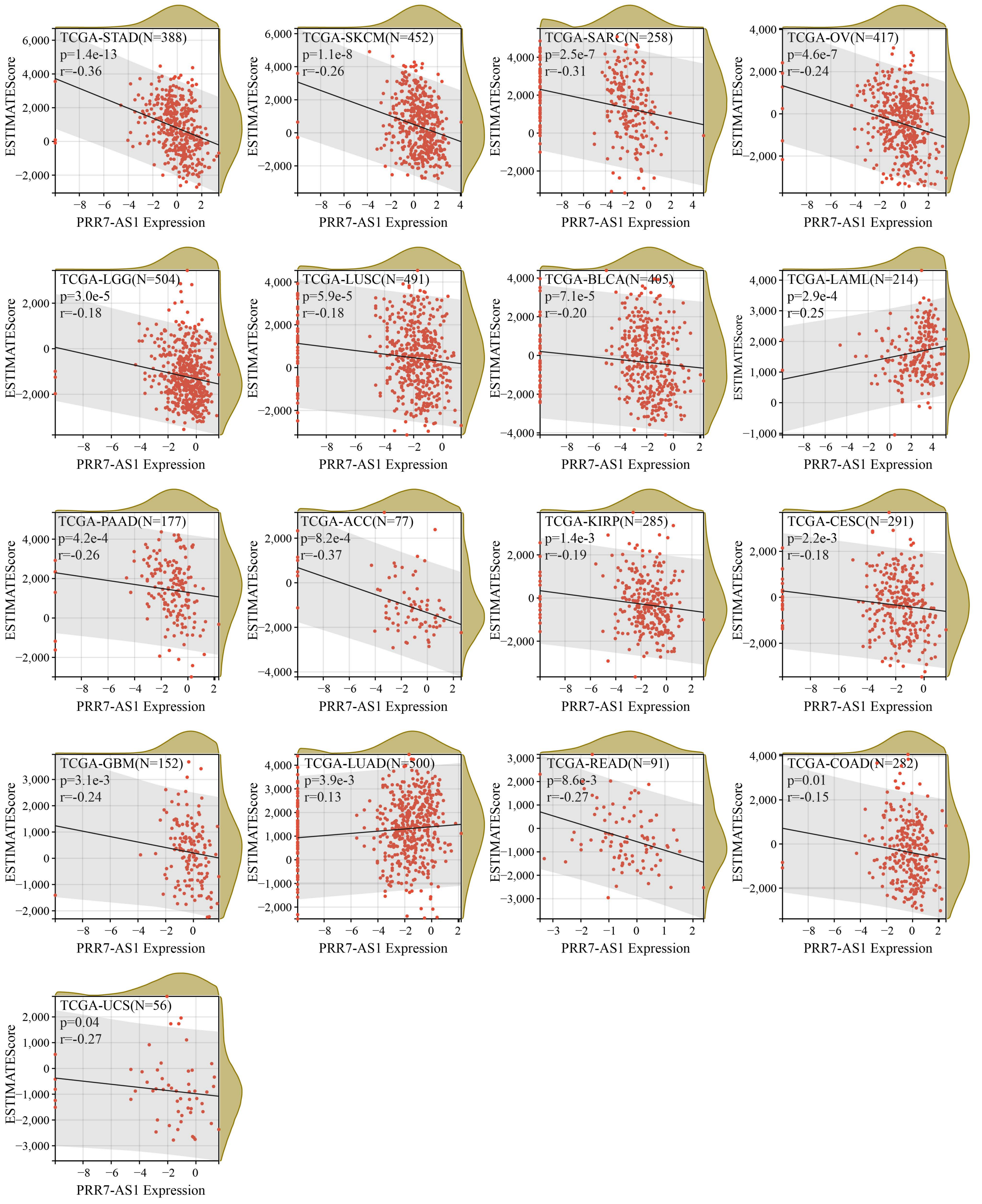

Supplementary Figure 6. Correlation between PRR7-AS1 expression levels and ESTIMATE score in pan-cancer. Correlation was tested by Spearman methods.

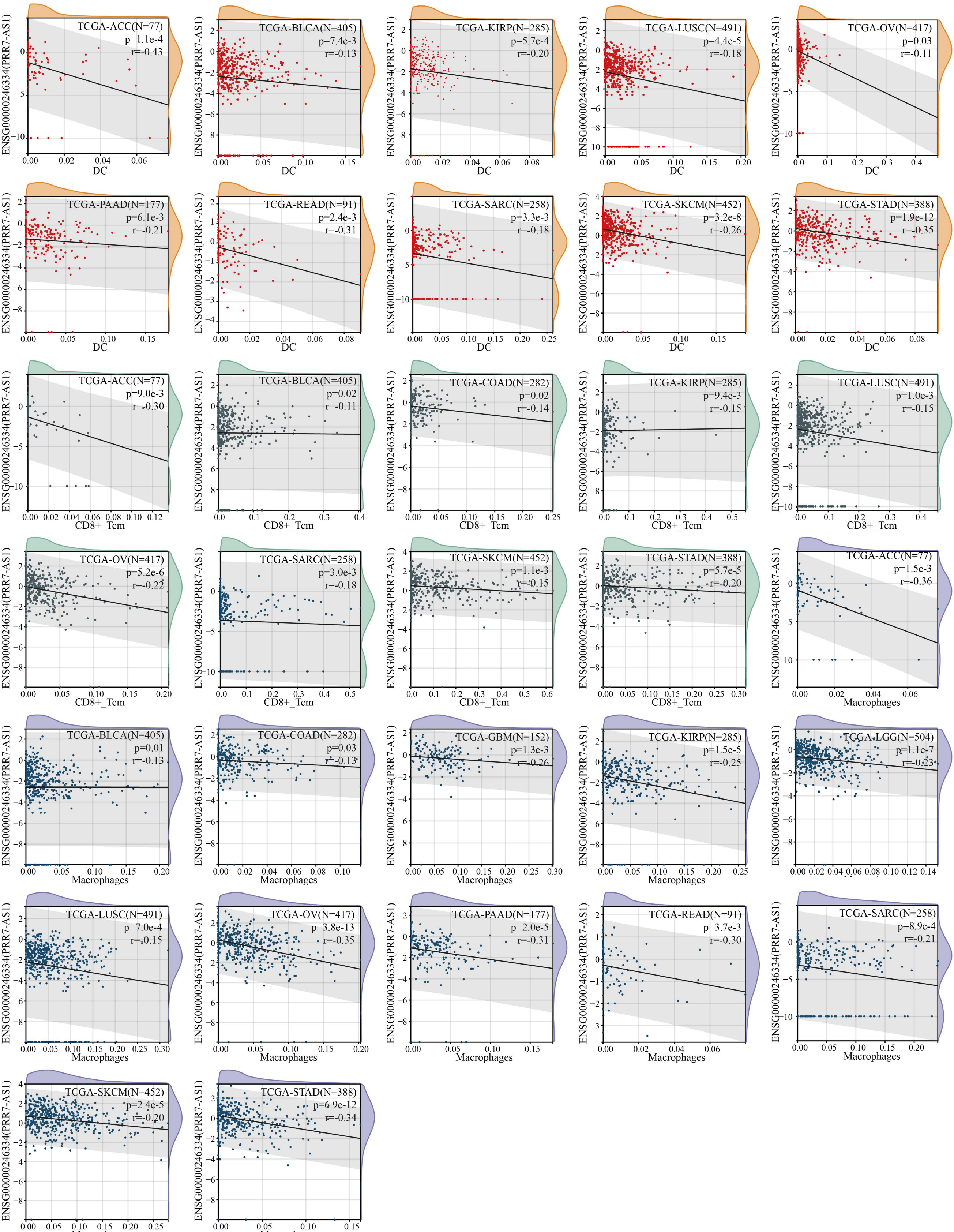

Supplementary Figure 7. Correlation analysis between PRR7-AS1 expression and immune cell infiltration. Spearman correlation between PRR7-AS1 expression and DC, CD8+ T and Macrophages in pan-cancer.

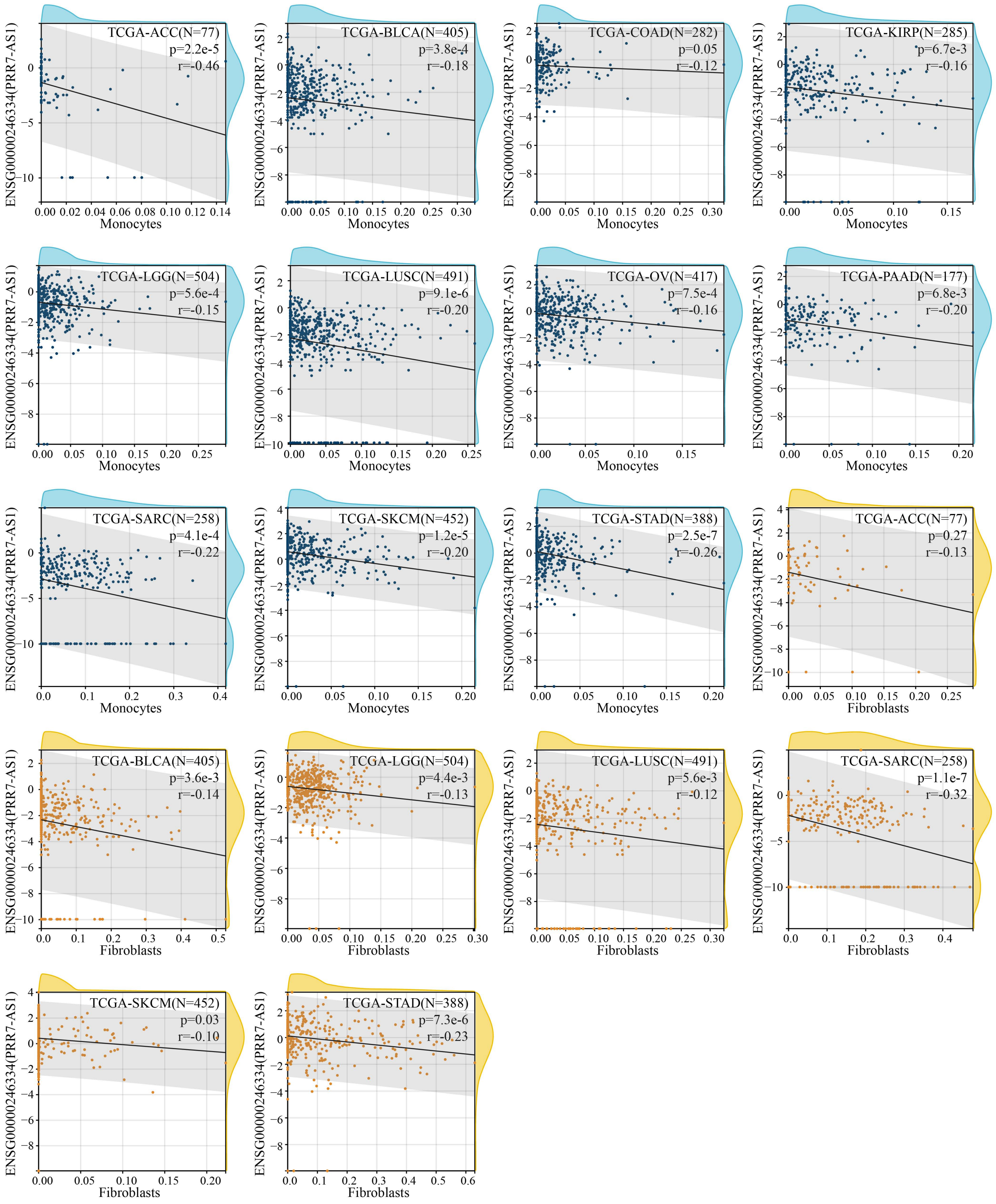

Supplementary Figure 8. Correlation analysis between PRR7-AS1 expression and immune cell infiltration. Spearman correlation between PRR7-AS1 expression and Monocytes and Fibroblasts in pan-cancer.

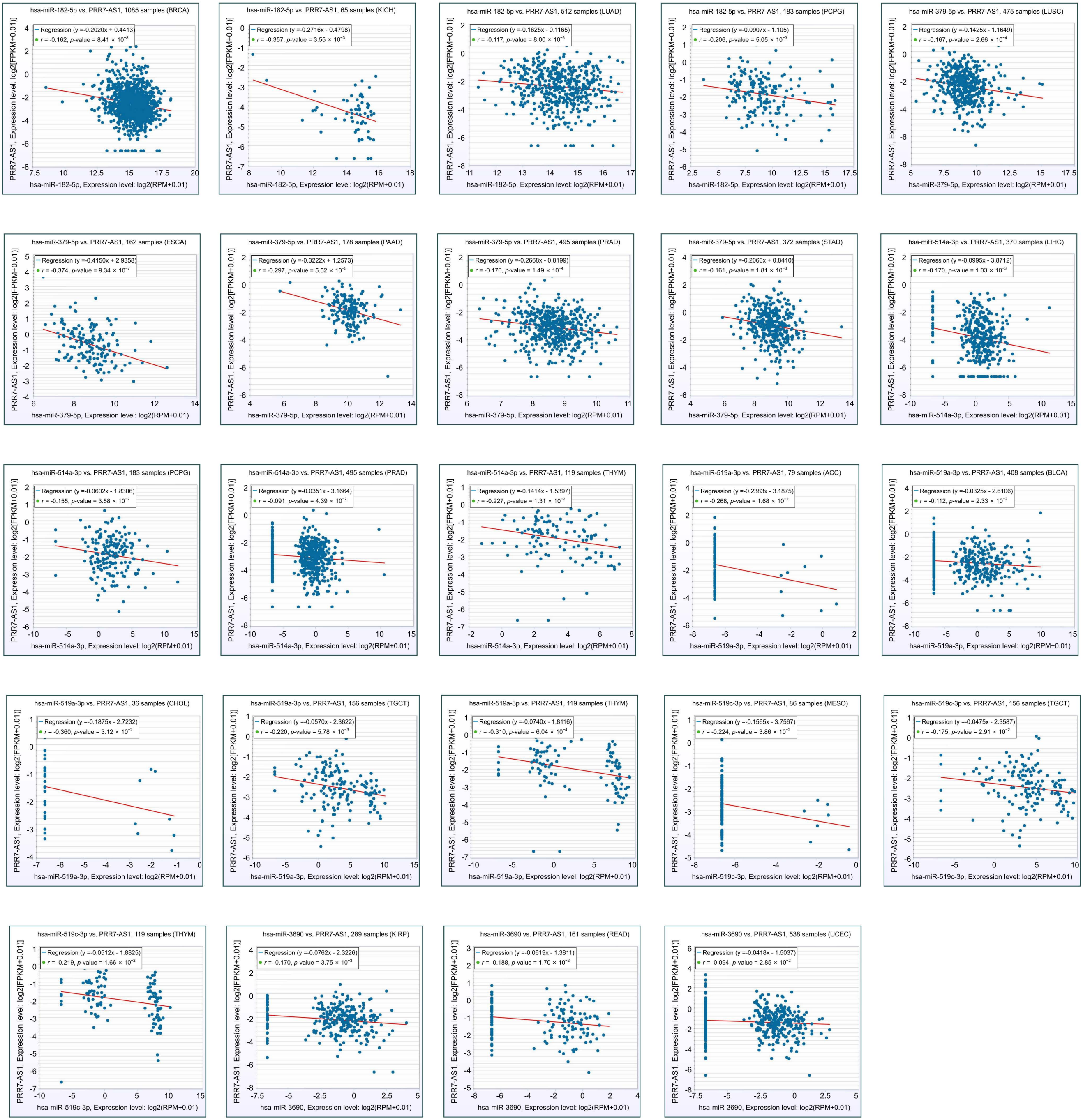

Supplementary Figure 9. Co-expression analysis for the miRNA-PRR7-AS1 interactions.

Table 1
List of abbreviations

Abbreviations	Full name
ACC	Adrenocortical carcinoma
BLCA	Bladder Urothelial Carcinoma
BRCA	Breast invasive carcinoma
CESC	Cervical squamous cell carcinoma and endocervical adenocarcinoma
CHOL	Cholangiocarcinoma
COAD	Colon adenocarcinoma
COADREAD	Colon adenocarcinoma/Rectum adenocarcinoma Esophageal carcinoma
DLBC	Lymphoid Neoplasm Diffuse Large B-cell Lymphoma
ESCA	Esophageal carcinoma
GBM	Glioblastoma multiforme
GBMLGG	Glioma
HNSC	Head and Neck squamous cell carcinoma
KICH	Kidney Chromophobe
KIPAN	Pan-kidney cohort (KICH+KIRC+KIRP)
KIRC	Kidney renal clear cell carcinoma
KIRP	Kidney renal papillary cell carcinoma
LAML	Acute Myeloid Leukemia
LGG	Brain Lower Grade Glioma
LIHC	Liver hepatocellular carcinoma
LUAD	Lung adenocarcinoma
LUSC	Lung squamous cell carcinoma
MESO	Mesothelioma
OV	Ovarian serous cystadenocarcinoma
PAAD	Pancreatic adenocarcinoma
PCPG	Pheochromocytoma and Paraganglioma
PRAD	Prostate adenocarcinoma
READ	Rectum adenocarcinoma
SARC	Sarcoma
STAD	Stomach adenocarcinoma
SKCM	Skin Cutaneous Melanoma
STES	Stomach and Esophageal carcinoma
TGCT	Testicular Germ Cell Tumors
THCA	Thyroid carcinoma
THYM	Thymoma
UCEC	Uterine Corpus Endometrial Carcinoma
UCS	Uterine Carcinosarcoma
UVM	Uveal Melanoma
CD274	CD274 molecule
CTLA4	Cytotoxic T-lymphocyte associated protein 4
DFI	Disease-free interval
DSS	Disease-specific survival

MSI	Microsatellite instability
OS	Overall survival
PDCD1	Programmed cell death 1
PD-L1	CD274 molecule
PFI	Progression-free interval
SEs	Super enhancers
TMB	Tumor mutational burden
TME	Tumor microenvironment
